# TDP-43 accelerates age-dependent degeneration of interneurons

**DOI:** 10.1038/s41598-017-14966-w

**Published:** 2017-11-02

**Authors:** Hitomi Tsuiji, Ikuyo Inoue, Mari Takeuchi, Asako Furuya, Yuko Yamakage, Seiji Watanabe, Masato Koike, Mitsuharu Hattori, Koji Yamanaka

**Affiliations:** 1 Department of Biomedical Science, Graduate School of Pharmaceutical Sciences, Nagoya City University, Nagoya, Aichi 467-8603 Japan; 2 Laboratory for Motor Neuron Disease, RIKEN Brain Science Institute, Wako, Saitama, 351-0198 Japan; 3 Department of Neuroscience and Pathobiology, Research Institute of Environmental Medicine, Nagoya University, Nagoya, Aichi 464-8601 Japan; 4 Department of Cell Biology and Neuroscience, Juntendo University Graduate School of Medicine, Bunkyo-ku, Tokyo, 113-8421 Japan

## Abstract

TDP-43 is an RNA-binding protein important for many aspects of RNA metabolism. Abnormal accumulation of TDP-43 in the cytoplasm of affected neurons is a pathological hallmark of the neurodegenerative diseases frontotemporal dementia (FTD) and amyotrophic lateral sclerosis (ALS). Several transgenic mouse models have been generated that recapitulate defects in TDP-43 accumulation, thus causing neurodegeneration and behavioural impairments. While aging is the key risk factor for neurodegenerative diseases, the specific effect of aging on phenotypes in TDP-43 transgenic mice has not been investigated. Here, we analyse age-dependent changes in TDP-43 transgenic mice that displayed impaired memory. We found the accumulation of abundant poly-ubiquitinated protein aggregates in the hippocampus of aged TDP-43 transgenic mice. Intriguingly, the aggregates contained some interneuron-specific proteins such as parvalbumin and calretinin, suggesting that GABAergic interneurons were degenerated in these mice. The abundance of aggregates significantly increased with age and with the overexpression of TDP-43. Gene array analyses in the hippocampus and other brain areas revealed dysregulation in genes linked to oxidative stress and neuronal function in TDP-43 transgenic mice. Our results indicate that the interneuron degeneration occurs upon aging, and TDP-43 accelerates age-dependent neuronal degeneration, which may be related to the impaired memory of TDP-43 transgenic mice.

## Introduction

Transactive response DNA binding protein of 43 kDa (TDP-43) is an RNA binding protein linked to the pathophysiology of neurodegenerative diseases such as amyotrophic lateral sclerosis (ALS) and frontotemporal dementia (FTD) (for review, see Renton *et al*. 2014^1^). ALS is characterized by progressive and selective neurodegeneration of motor neurons, while FTD is characterized by progressive neuronal loss predominantly in the frontal and/or temporal lobes. For both ALS and FTD, a fraction of the patients develop the inherited form of the disease. Recent research has mainly focused on hereditary cases, whose causative genes have been identified (e.g., *SOD1* and *C9orf72* for ALS, and *C9orf72, MAPT*, and *progranulin* for FTD). In this study, we focus instead on the sporadic form of the disease, which represents 90% of ALS cases and 60% of FTD cases. Abnormal accumulation of hyper-phosphorylated and poly-ubiquitinated TDP-43 protein has been found in the affected neurons in nearly half of all FTD cases and in 97% of the ALS cases^2–4^. While TDP-43 accumulates in the cytoplasm in most cases, it can also aggregate in the nucleus in some cases. It is thought that the dysregulation of TDP-43 causes neuronal dysfunction, subsequently leading to neuronal degeneration.

TDP-43 is involved in various aspects of RNA metabolism including pre-mRNA splicing, transport of RNA granules, and the formation of ribonucleoprotein granules^4,5^. Genome-wide analyses of TDP-43 RNA binding targets reveal that TDP-43 can bind to thousands of messenger RNAs (mRNAs)^6,7^. TDP-43 regulates pre-mRNA splicing of some genes, including the cystic fibrosis transmembrane receptor gene^8^ and its own mRNA^9^ by stimulating or inhibiting alternative exon inclusion. TDP-43 shuttles from the nucleus to the cytoplasm and is thought to have a function in the cytoplasm as well as in the nucleus, perhaps in the regulation of neuronal RNA granule transport along axons and dendrites. Importantly, beyond the pathological TDP-43 inclusions seen in the sporadic disease form, several mutations in TDP-43 have been identified as a cause of some familial and sporadic ALS and FTD cases^4,10^, further emphasizing the critical role of TDP-43 in the pathogenesis of ALS/FTD. However, whether these mutations and pathological aggregation of TDP-43 cause disease by a loss of function, gain of function, or some combination of both remains unresolved^11^.

In patients with sporadic ALS/FTD, the levels of TDP-43 mRNA and protein are elevated by about 1.5-fold^12^ and 1.5–2.5-fold, respectively, in affected brain regions^13,14^. Several reports indicate that elevated levels of wild-type TDP-43 are sufficient to cause neurological and pathological phenotypes mimicking FTD/ALS in mice^15–17^. Therefore, transgenic (Tg) mice expressing elevated levels of wild-type TDP-43 are appropriate disease models to capture the pathology of sporadic ALS/FTD in mice.

In this study, to define the pathomechanisms of sporadic ALS/FTD and to investigate the contribution of aging to the formation of such phenotypes, we generated Tg mice expressing wild-type TDP-43 under the control of the mouse prion promoter and defined the pathology, behaviour, and genes affected by the dysregulation of TDP-43 during aging. Consistent with other TDP-43 models, the Tg mice developed learning and memory deficits as well as mild impairment of motor function^15,18^. Interestingly, we observed massive aggregates derived from GABAergic inhibitory interneurons in the hippocampus of TDP-43 Tg mice. Intriguingly, we also observed the aggregates in aged wild-type mice; the aggregates increased as the animals got older, indicating that the degeneration of GABAergic interneurons occurs during aging and is accelerated by the increased accumulation of TDP-43.

## Results

### Generation and characterization of TDP-43 transgenic mice

**Figure 1:**
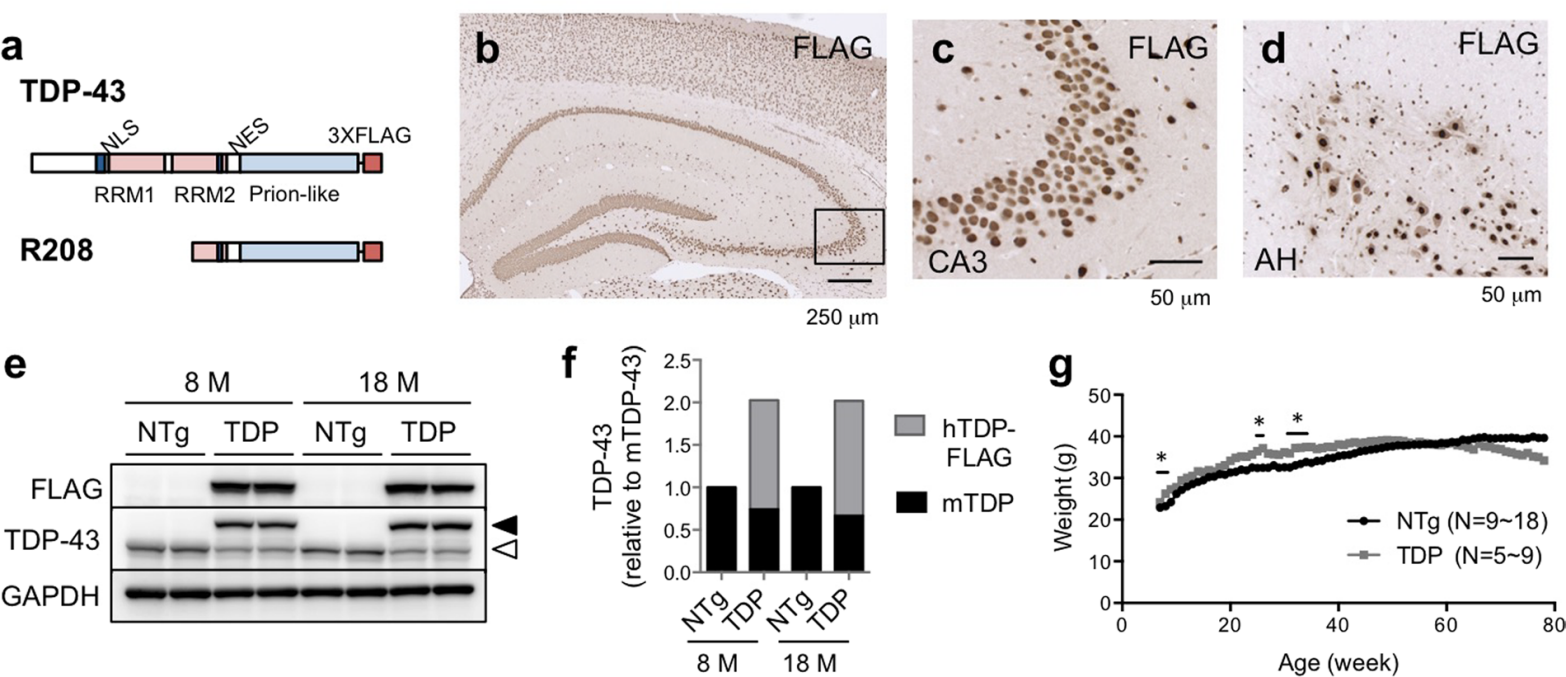
Generation and characterization of TDP-43 Tg mice. (**a**) Schematic diagram of FLAG-tagged TDP-43 and FLAG-tagged TDP-43 C-terminal fragment (R208, amino acid residue 208 to 414), which is the accumulated form of TDP-43 in the affected regions of patients with FTD/ALS, used to generate the Tg mice under the control of the mouse prion promoter. (**b–d**) Immunostaining of FLAG-tagged TDP-43 in TDP-43 Tg mice. The sections of the hippocampus (**b**), CA3 region of the hippocampus (**c:** the magnified image in the boxed region of Fig. 1b), and the anterior horn of the spinal cord (**d**) of 8-month-old TDP-43 Tg mice were stained with the anti-FLAG antibody. (**e**) Immunoblots of brain tissues of non-Tg littermate (NTg), heterozygous TDP-43 Tg mice (TDP) at 8 months and 18 months of age. Whole brain tissues of 8-month-old mice with the indicated genotypes were immunoblotted with anti-TDP-43, anti-FLAG, or anti-GAPDH antibodies. The filled and open arrowheads denote hTDP-43 (transgene) and mTDP-43 (endogenous), respectively. (**f**) Quantification of the amount of TDP-43 relative to wild-type TDP-43 in NTg mice. The intensities of bands shown in Fig. 1E were quantified using the MultiGauge software; n = 2. (**g**) Body weights of TDP-43 Tg mice (TDP) and non-Tg mice (NTg). Averaged body weights of the male mice with the indicated genotypes were plotted. Unpaired t-test, *p < 0.05.

To recapitulate the pathology of sporadic ALS/FTD in mice, we generated transgenic (Tg) mice in which full-length wild-type human TDP-43 was expressed under the control of the mouse prion promoter. We also generated mice expressing a truncated form of TDP-43, containing the C-terminal region (amino acid residues 208–414: R208) (Fig. 1a). This C-terminal fragment of TDP-43 is abundantly found in the affected neurons in patients with ALS/FTD^19^. We confirmed expression of FLAG-tagged TDP-43 by immunohistochemistry using an anti-FLAG antibody (Fig. 1b–d). The FLAG-tagged TDP-43 was localized mainly in the nuclei of neurons in the brain and spinal cord. We did not observe cytoplasmic accumulation of TDP-43 in the brain or spinal cord of the TDP-43 Tg mice even at 18 months of age (data not shown).

To assess the expression levels of TDP-43 in the brain tissue, we performed western blot analysis. The mean level of FLAG-tagged human TDP-43 in 8-month-old heterozygous TDP-43 Tg mice (TDP) was 1.28 fold higher than the endogenous mouse TDP-43 level in non-transgenic (NTg) mice (Fig. 1e,f). TDP-43 is known to bind to the 3’ UTR of its own mRNA to induce its degradation, via an auto-regulation mechanism^20^. Consistent with this, quantitative analysis of the western blotting data revealed that the overexpression of human TDP-43-FLAG (Fig. 1e, filled arrowhead) reduced the level of endogenous mouse TDP-43 protein (Fig. 1e, open arrowhead) to 74.1% (Fig. 1e,f) of its original level. Therefore, the mean total TDP-43 (endogenous and exogenous TDP-43) in the brains of 8-month-old TDP-43 Tg mice was 2.03 times that of the mean total endogenous TDP-43 in the NTg animals (Fig. 1e,f). These ratios were not significantly different in the brains of 18-month-old mice (Fig. 1e,f; FLAG-tagged human TDP-43, 1.35-fold; endogenous mTDP-43, 0.67-fold; total TDP-43, 2.02-fold). In the transgenic mice expressing the C-terminal TDP-43 fragment R208 (R208 Tg mice), the FLAG-tagged R208 level was 12.6% of the FLAG-tagged TDP-43 level in TDP-43 Tg mice (Supplementary Fig. S1a, n = 4). As previously reported, the endogenous mouse TDP-43 expression was not affected in the presence of the C-terminal TDP-43 protein (Supplementary Fig. S1b), given that R208 lacks an RNA-binding motif and has no ability to autoregulate its mRNA levels^9^.

The mean body weight of the TDP-43 Tg mice was slightly higher than that of the non-Tg mice at 7–9, 25, 26, and 30–35 weeks of age. While the heterozygous TDP-43 Tg mice showed a trend of a decrease in body weight with increasing age, they did not show obvious signs of paralysis for over 2 years.

To investigate the effect of further increases in the expression of TDP-43, we crossed male and female heterozygous TDP-43 Tg mice to generate homozygous TDP-43 Tg mice (TDP/TDP). All the homozygous progeny died between postnatal (P) days 25 and 27 with severe paralysis of the limbs (Supplementary Fig. S2c, Supplementary Movie S1). This result is consistent with that observed by Wils *et al*., who used the Thy1 promoter to drive the TDP-43 expression^17^. At P20, the levels of exogenous hTDP-43-FLAG protein in the heterozygous and homozygous progeny were 74% and 112% of the endogenous mouse TDP-43 protein levels in NTg mice, respectively (Supplementary Fig. S2a,b). The endogenous expression of TDP-43 was only slightly affected (Supplementary Fig. S2a,b; TDP/-, 86%; TDP/TDP, 84%). Therefore, the total amount of TDP-43 in the heterozygous and homozygous progeny was 1.6-fold and 2-fold of that in NTg mice, respectively. Despite this modest increase in the total TDP-43 levels in the homozygous progeny compared with the heterozygous mice, homozygous progeny (TDP/TDP) displayed microglial activation in the spinal cords in addition to muscle fibre atrophy and degeneration (Supplementary Fig. S2d). These data indicated that there might be a threshold amount beyond which TDP-43 becomes extremely toxic to the central nervous system and muscles.

### Memory deficits in TDP-43 transgenic mice

**Figure 2:**
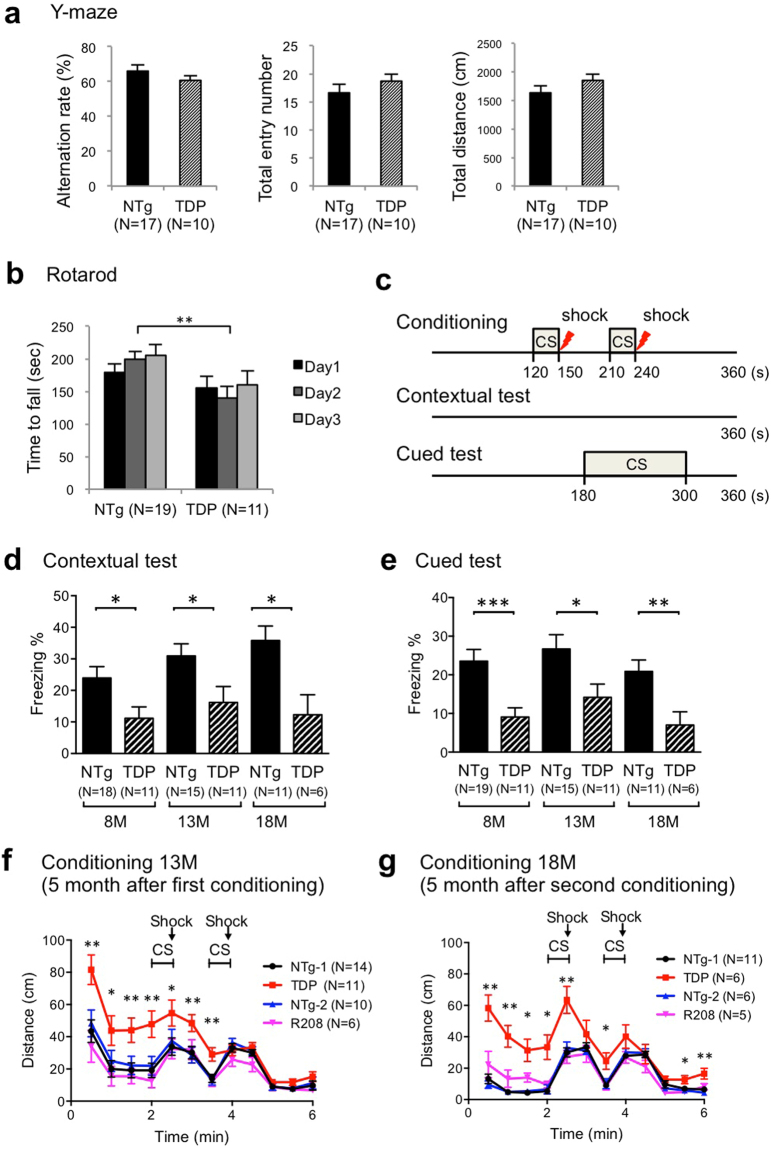
Mild impairment in motor learning and severe memory deficit in heterozygous TDP-43 Tg mice. (**a**) Quantitative analysis of the Y-maze test data. The mean alteration rate, total number of entries, and total distance travelled by non-Tg (NTg) and TDP-43 Tg (TDP) mice are plotted. (**b**) Quantitative analysis of the rotarod test data. The mean holding time on the rotating rod at 8 months of age over three sequential trials is shown. (**c**) Experimental design used for contextual and cued fear conditioning tests. (**d–g**) Quantitative analysis of contextual and cued fear conditioning test data. The percentage of mean freezing times in the contextual test (**d**) and cued test (**e**) at the indicated ages is plotted. The distances travelled during conditioning of 13-month-old mice (**f**) or 18-month-old mice (**g**) are plotted for each genotype. Unpaired t-test; the data are presented as mean ± SEM. Significance level thresholds of *p < 0.05, **p < 0.01, and ***p < 0.001 were used (Fig. 3a,b,d–g).

To investigate whether abnormal TDP-43 accumulation causes the cognitive phenotype related to FTD/ALS in mice, we performed serial behavioural tests including the Y-maze test, rotarod test, and contextual and cued fear conditioning tests. First, to assess the working memory and activity in heterozygous TDP-43 Tg mice, we performed the Y-maze test. There was no significant difference between the TDP-43 Tg (TDP) and non-Tg (NTg) mice at 8 months of age in either the alternation rate or the total number of entries (Fig. 2a, left and middle), indicating that the working memory of the TDP-43 Tg mice was normal. The total distance travelled by the TDP-43 Tg mice was also similar to that travelled by the non-Tg mice, indicating that the activity was also normal in the TDP-43 Tg mice (Fig. 2a, right). The same mice were tested on the Y-maze test when they were at 13 months and 18 months of age, and there was no significant difference between the TDP-43 Tg and non-Tg mice in the alternation rate, total number of entries, or the total distance travelled, suggesting that aging did not affect short-term memory and activity in TDP-43 Tg mice (Supplementary Fig. S3a,b). These data showed that the short-term memory and activity are both normal in TDP-43 Tg mice.

Next, we performed a rotarod test to analyse motor function and learning. There were statistically significant differences between TDP-43 Tg and non-Tg mice (age, 8 months) in the latency to fall on day 2 (Fig. 2b; Day 2, *p* = 0.0088). The test was conducted when the mice were 13 and 18 months old; 13-month-old mice showed a shorter latency to fall (Supplementary Fig. S3c; Day 1, *p* = 0.0047; Day 2, *p* = 0.0448; Day 3, *p* = 0.0203). These results indicate that motor function and learning were moderately impaired in the TDP-43 Tg mice.

Finally, to assess associative fear learning and memory in mice, we performed contextual and cued fear conditioning tests. In the contextual test, there was a significant difference between the TDP-43 Tg mice (TDP) and non-Tg mice (NTg) in the percentage of freezing time at all the ages tested, indicating that TDP-43 Tg mice displayed defective contextual fear memory (Fig. 2d; 8 M, *p* = 0.0118; 13 M, *p* = 0.0311; 18 M, *p* = 0.013). In the cued test, there was also a significant difference between the TDP-43 Tg mice (TDP) and non-Tg mice (NTg) in the percentage of freezing time at all the ages tested (Fig. 2e; 8 M, *p* = 0.0009; 13 M, *p* = 0.0206; 18 M, *p* = 0.0099), indicating that TDP-43 Tg mice had impaired cued fear memory formation.

We further noticed that the distance travelled during the conditioning of mice at 13 months of age was significantly longer in TDP-43 Tg mice than in non-Tg mice (NTg-1) (Fig. 2f). Likewise, the distance travelled during the conditioning of mice at 18 months of age was significantly longer in TDP-43 Tg mice than in non-Tg mice (NTg-1) (Fig. 2g). Therefore, the long-term memory might be impaired in TDP-43 Tg mice.

In heterozygous R208 Tg mice, there was no significant difference between NTg mice and R208 mice in all the tests performed (Fig. 2f,g, Supplementary Fig. S4). Therefore, overexpression of the C-terminal fragment, the accumulated form of TDP-43 in patients, was not sufficient to induce memory deficits in mice. Alternatively, the lack of a phenotype in R208 Tg mice may be due to low level of expression of this fragment of TDP-43, as shown in Supplementary Fig. S1.

### Poly-ubiquitin- and p62-positive aggregates derived from GABAergic interneurons in the hippocampus of aged mice

**Figure 3:**
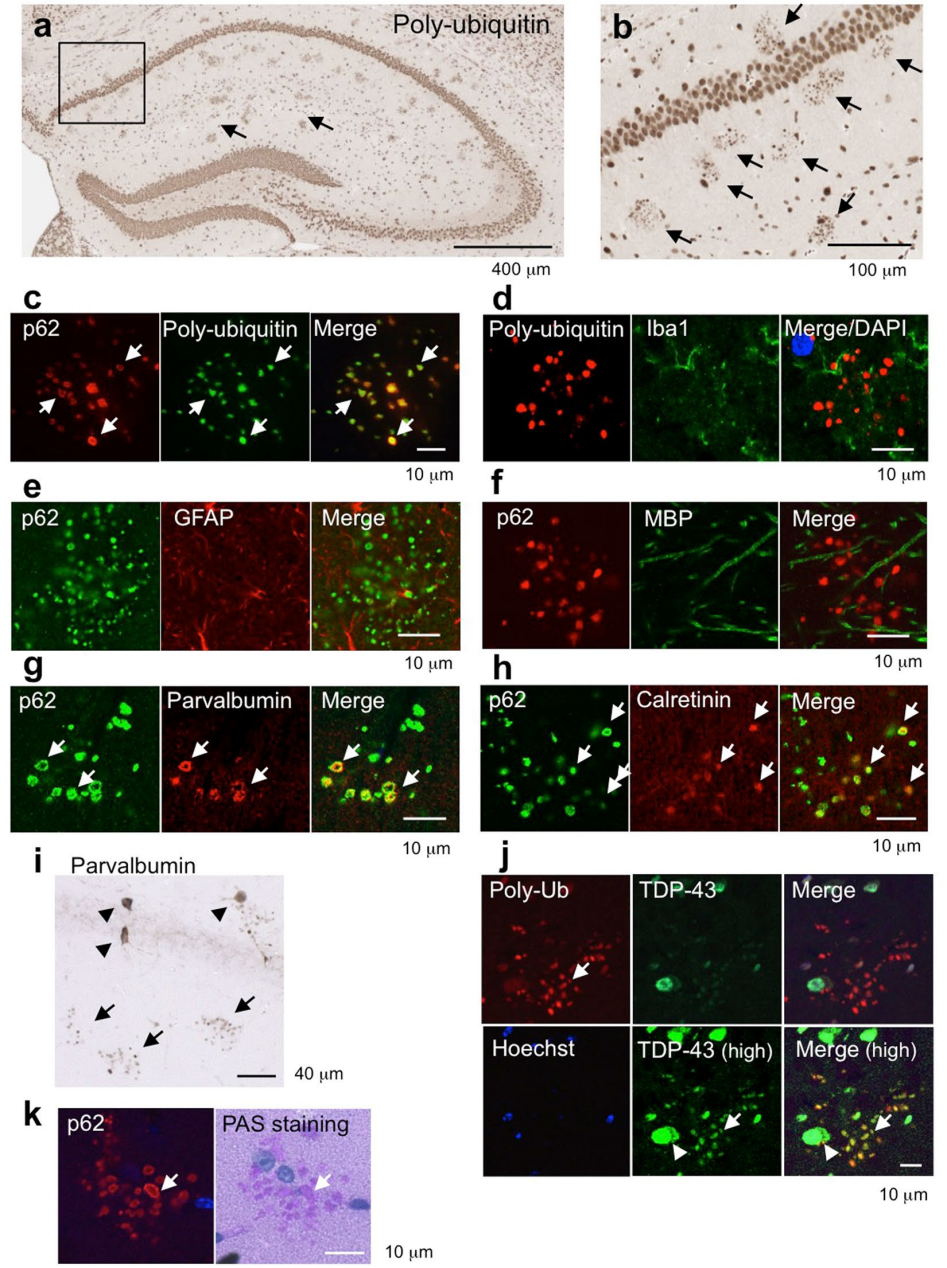
Massive poly-ubiquitin- and p62-positive aggregates derived from GABAergic interneurons in the hippocampus of aged wild-type mice and TDP-43 Tg mice. (**a**,**b**) Immunostaining of poly-ubiquitin in the hippocampus of non-Tg mice at 20 months of age. The magnified image in the boxed region of Fig. 1a is shown (**b**). The arrows indicate colonies of poly-ubiquitin-positive aggregates. (**c**–**h**) Immunofluorescence staining of poly-ubiquitin- and p62-positive aggregates with cell type-specific markers. The arrows indicate co-localization. The aggregates contained poly-ubiquitin and p62 (**c**), but not glial markers Iba1 (**d**), GFAP (**e**), or MBP (**f**). The aggregates contained parvalbumin (**g**) and calretinin (**h**), indicating that these are the aggregates derived from GABAergic interneurons. (**i**) DAB staining of the hippocampus of TDP-43 Tg mice with anti-parvalbumin (PV) antibody. The arrowheads denote intact PV neurons, while arrows denote aggregates derived from PV neurons. (**j**) Immunofluorescence staining of aggregates in the hippocampus of aged mice with anti-p62 and anti-TDP-43 antibodies. The arrows indicate co-localization. The arrowhead indicates cytoplasmic TDP-43. (**k**) Immunofluorescence staining of p62-positive aggregates following PAS staining of the hippocampus of aged mice.

We further investigated the histopathological changes in the brains of TDP-43 Tg mice. Intriguingly, we found numerous poly-ubiquitin-positive aggregates in the hippocampus of both TDP-43 Tg mice and aged non-Tg mice (Fig. 3a,b). The aggregates were also immunoreactive for p62 (Fig. 3c). This pattern indicates that selective autophagy was impaired. Normal mouse Ig was used to confirm the specificity of mouse monoclonal antibodies (Supplementary Fig. S5b).

To analyse whether these aggregates were derived from glial cells, we stained brain sections with antibodies against GFAP, Iba1, or Mac2. We did not observe these cell types to co-stain with p62 or poly-ubiquitin, indicating that the aggregates were not located in glial cells (Fig. 3d–f). In contrast, the aggregates stained positive for GABAergic inhibitory interneuron markers, parvalbumin (PV) and calretinin (CR) (Fig. 3g,h). Since the size of an aggregate was about 1–2 μm (diameter), these were not intact interneurons (Fig. 3i, arrowheads, intact cells; arrow, aggregates). Tau, which stabilizes microtubules and is observed to abnormally accumulate in Alzheimer’s disease, was also a component of the aggregates (Supplementary Fig. S5f). Since we did not detect phosphorylated Tau, recognized with AT8 or AT180 antibodies, in the aggregates (data not shown), the Tau present in the aggregates may be a non-phosphorylated form. MAP2, another microtubules-stabilizing protein localized in cell bodies and dendrites, was also not a component of the aggregates (Supplementary Fig. S5g). Moreover, anti-TDP-43 antibody did not stain the aggregates strongly, suggesting that TDP-43 was not a major component of these aggregates (Fig. 3j). However, we observed very weak staining in the aggregates at the intensity similar to that of cytoplasmic TDP-43 (Fig. 3j, high sensitivity), suggesting that a very small amount of TDP-43 was included in the aggregates.

We have also found that the Periodic-acid-Schiff (PAS) staining in the brains of aged mice showed a similar cluster pattern (Fig. 3k, Supplementary Fig. S5a). A PAS-positive granule was first described as a granular structure increased in the brains of senescence-accelerated mice (SAM)^21,22^. We immunostained the aggregates with anti-p62 antibody following PAS-staining, which confirmed that these were the same granules (Fig. 3k). Sometimes, we observed negative staining of p62 in the center of aggregates, but it was always stained with PAS (Fig. 3k, arrow).

**Figure 4:**
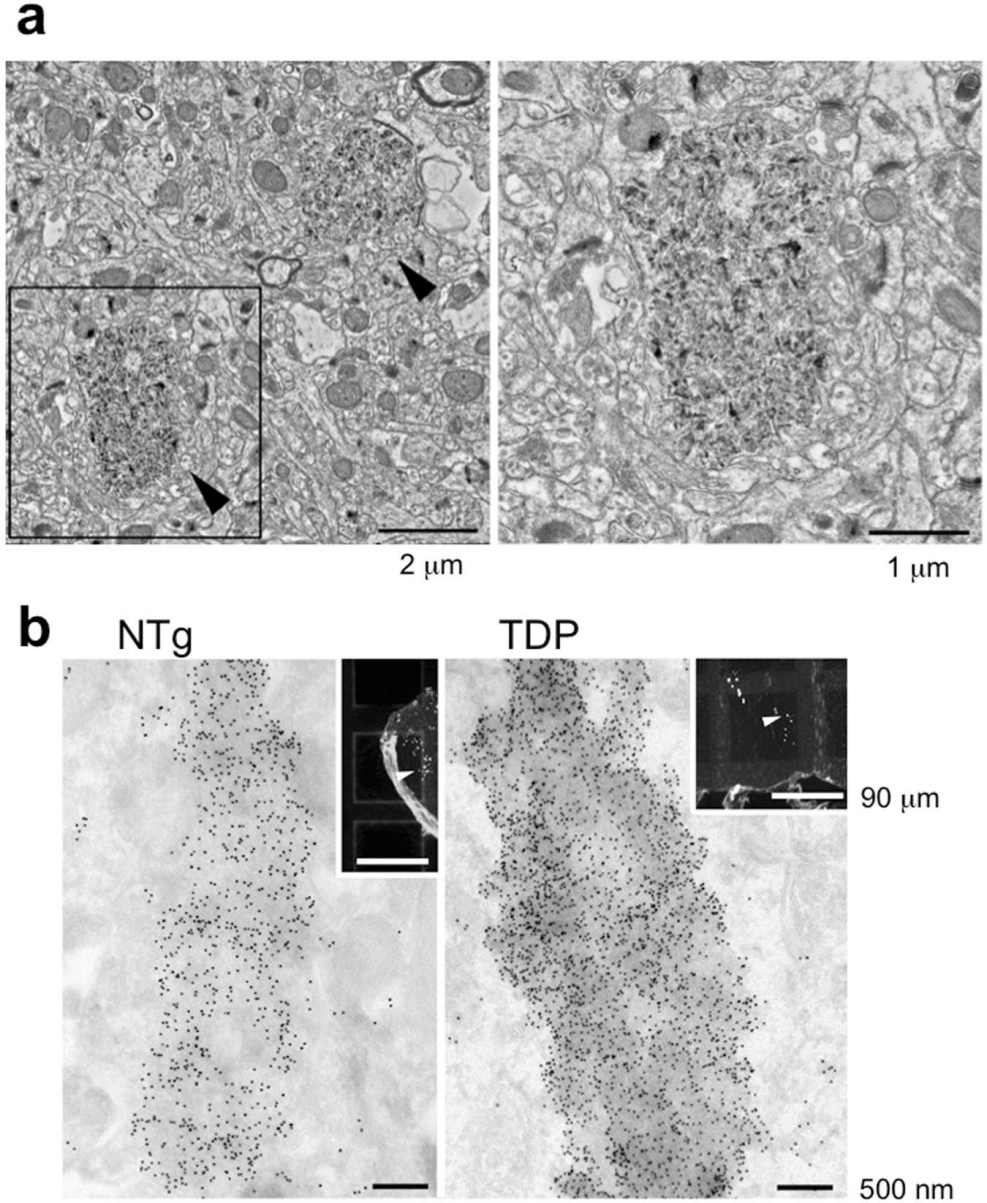
Ultrastructural analyses of the cytoplasmic aggregates of the aged mice. (**a**) Conventional electron microscopy of the CA1 part of stratum radiatum of the hippocampus of the aged non-Tg mice. A boxed area in the left panel is enlarged and shown in the right panel. The cytoplasmic aggregates surrounded by a plasma membrane are about 1-2 µm in diameter and composed of electron-dense crystalline-like fibrillary structures. Bars: 2 µm (left) and 1 µm (right). (**b**) Correlative light- and immunoelectron microscopy on Tokuyasu cryosections of the hippocampus of the TDP-43 Tg (left) and aged non-Tg mice (right). 10 nm-gold particles indicating p62 were massively accumulated in the cytoplasmic aggregates corresponding to those with coarse granular fluorescence for p62 (arrows in insets). Bars: 500 nm and 90 µm (insets).

Electron microscopy of the hippocampus of aged mice revealed that the aggregates were about 1-2 µm in diameter and composed of electron-dense crystalline-like fibrillary structures. Importantly, the aggregates were surrounded by a plasma membrane, suggesting their intracellular location (Fig. 4a).

Furthermore, by correlative light- and immunoelectron microscopy on Tokuyasu cryosections of the hippocampus of the TDP-43 Tg and aged non-Tg mice we confirmed that massive gold particles indicating p62 were accumulated in the cytoplasmic aggregates corresponding to those with coarse granular fluorescence for p62 (Fig. 4b). PAS, ubiquitin and p62-positive cytoplasmic aggregates in the parvalbumin neurons shared the similarity for polyglucosan bodies known as corpora amylacea^23^ or PAS granules^24^ observed in the brain of aged mice.

### Increased number of aggregates derived from GABAergic interneurons in TDP-43 Tg mice

**Figure 5:**
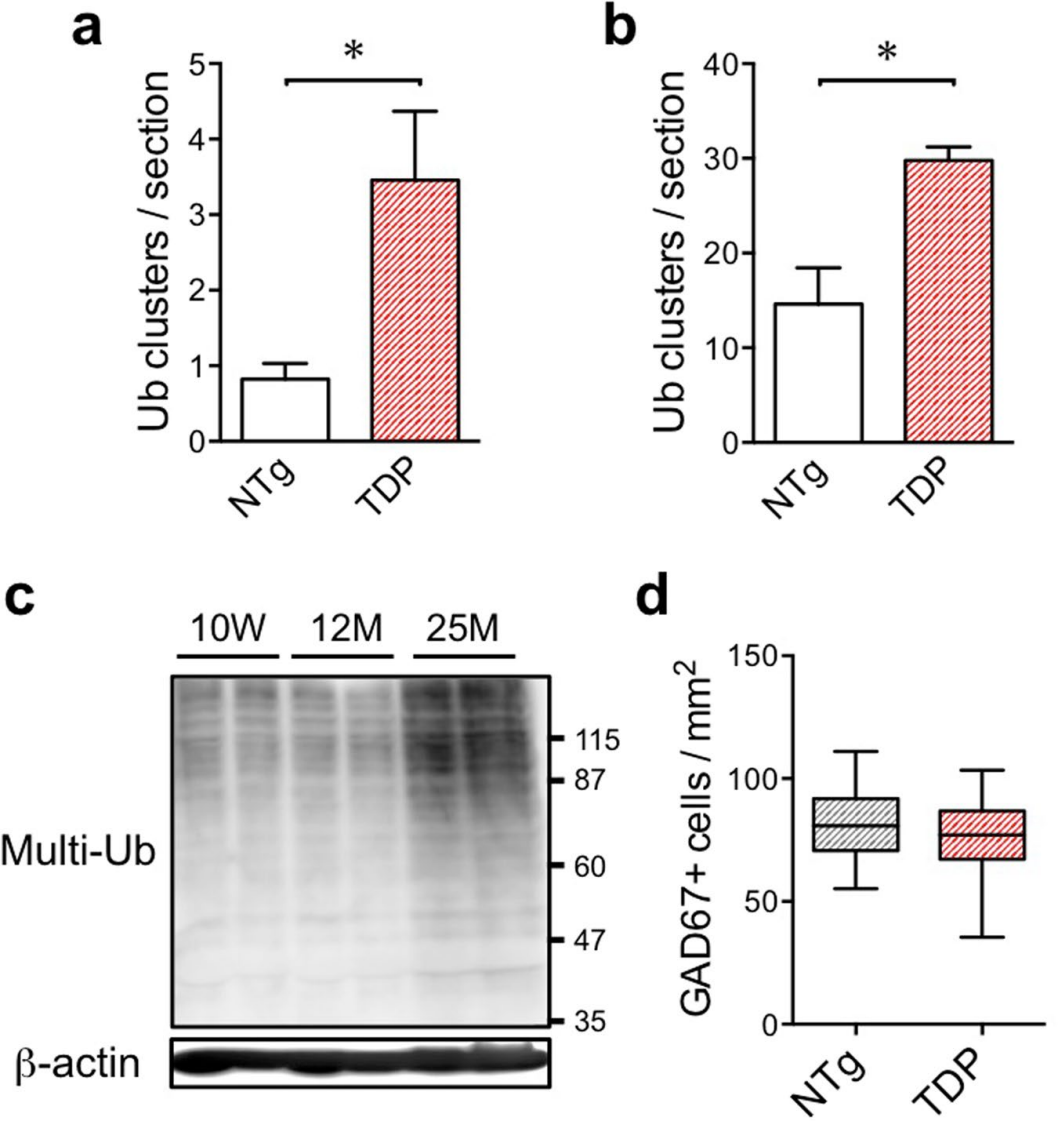
The number of aggregates derived from GABAergic interneurons are increased in TDP-43 Tg mice. (**a**) The averaged number of poly-ubiquitin clusters per hippocampal section of NTg and TDP-43 Tg mice at 8 months of age is plotted (mean of NTg, 0.7; mean of TDP, 2.6). The clusters bigger than 20 μm in diameter were counted. The averages of clustered number for each mouse are plotted and statistically analysed between NTg and TDP. More than 5 sections with the hippocampus bigger than 0.6 mm^2^ were used for each mouse. The numbers of sections used for quantification were as follows: NTg, 75 sections from 8 mice; TDP-43 Tg, 71 sections from 7 mice. Unpaired t test, *p < 0.05. (**b**) The averaged number of poly-ubiquitin clusters per hippocampal section of NTg and TDP-43 Tg mice at 20 months of age is plotted. The clusters bigger than 50 μm in diameter were counted. The averages of clustered number for each mouse are plotted and statistically analysed between NTg and TDP. More than 7 sections with the hippocampus bigger than 0.6 mm^2^ were used for each mouse. The numbers of sections used for quantification were as follows: NTg, 102 sections from 5 mice; TDP-43 Tg, 91 sections from 4 mice. Unpaired t test, *p < 0.05. (**c**) The increase in poly-ubiquitinated proteins in RIPA-insoluble fraction of the hippocampus of aged mice compared with those of young mice. RIPA-insoluble fractions of the hippocampus of indicated age were blotted with anti-multi-ubiquitin antibody. The same membrane was restained with anti-β-actin antibody. (**d**) Number of GAD67-positive neurons in the hippocampus on the section adjacent to the section used in Fig. 4j. The numbers of sections used for quantification were as follows: NTg, 44 sections from 3 mice; TDP-43 Tg, 68 sections from 3 mice.

Interestingly, the numbers of these poly-ubiquitin-positive aggregates are higher in TDP-43 Tg mice than in NTg mice at 8 months of age (Fig. 5a, *p* < 0.05) and 20 months of age (Fig. 5b, *p* < 0.05), indicating an increased number of aggregates derived from GABAergic interneurons in TDP-43 Tg mice. To confirm the increase of multi-ubiquitinated proteins in aged mice, the hippocampus of aged mice were analysed by western blot analysis (Fig. 5c). The amount of multi-ubiquitinated proteins in RIPA-insoluble fraction was increased upon aging.

To analyse whether the number of interneurons was lower in TDP-43 Tg mice due to accelerated neuronal degeneration, we counted GAD67-positve interneurons using sections adjacent to those used to count the p62-positve aggregates. Representative images of the sections used for counting are shown in Supplemental Fig. 5e. There was no significant difference between TDP-43 Tg mice and non-Tg mice in the number of GAD67-positive interneurons in the hippocampus of 20-month-old mice (Fig. 5d).

### Differentially expressed or alternatively spliced genes in TDP-43 Tg mice

**Figure 6:**
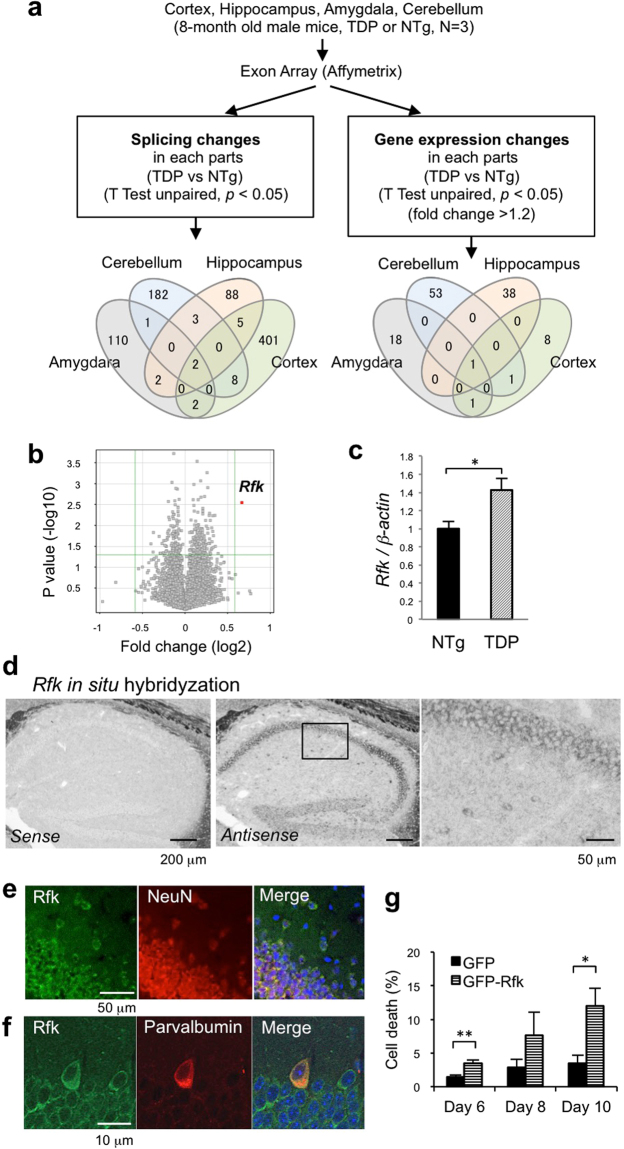
Increased expression of riboflavin kinase in the hippocampus of TDP-43 Tg mice. (**a**) Experimental design of the exon array analysis. The numbers in the Venn diagram indicate the number of genes identified. (**b**) Plots of the expression levels of genes in the hippocampus of TDP-43 Tg mice compared with those in NTg mice. A red spot indicates the *Rfk* gene. (**c**) Quantitative PCR analysis of *Rfk* mRNA in the hippocampus of TDP-43 Tg mice and NTg mice. (Unpaired t-test, *p < 0.05) n = 3 each. (**d**) Representative images of *Rfk in situ* hybridisation using hippocampal sections of 8-month-old wild-type mice. Signals obtained with sense (left) and anti-sense probes (middle, right) are shown. The magnified image in the boxed region of the middle panel is shown (right). (**e**) Immunostaining of Rfk and NeuN in the hippocampus of 8-month-old non-Tg mice. (**f**) Immunostaining of Rfk and parvalbumin (PV) in the hippocampus of 8-month-old non-Tg mice. (**g**) Primary cultured hippocampal neurons were transfected with GFP or GFP-Rfk on day 3 of culture after dissociation of the hippocampus, and the cells were fixed and co-stained with GFP and CC3 antibodies to assess cell death. The percentage of CC3-positive cells among the total number of GFP-positive cells was calculated. The data from three independent experiments are combined and plotted. More than 200 cells for each column were counted, and more than 600 cells were counted for day 6. The data are presented as mean ± SEM. Unpaired t-test, *p < 0.05, **p < 0.01.

To define the molecules that might be involved in interneuron degeneration and pathogenesis of TDP-43 Tg mice, we analysed changes in gene expression and alternative splicing in TDP-43 Tg mice. We isolated the hippocampus, cerebral cortex, amygdala, and cerebellum from 8-month-old mice (n = 3). We extracted RNA from these brain regions, hybridized the RNAs to an exon array, and analysed for changes in splicing and gene expression (Fig. 6a). We observed significant splicing differences (*p* < 0.05, splicing index > 0.5) in 804 genes in either one of the tissues between TDP-43 Tg mice and NTg mice. We further analysed the microarray results for any overlaps between brain regions, and found that only a small numbers of genes overlapped (Fig. 6a, left). Significant gene expression changes (*p* < 0.05, 1.2-fold change) in either one of the brain regions of TDP-43 Tg mice compared with those of NTg mice were observed in 122 genes (Supplementary Table S1). Several genes were upregulated in TDP-43 mice, including genes involved in neuronal function or oxidative stress. We further analysed these genes for overlaps between brain regions, and again, only a small number of genes were seen to overlap (Fig. 6a, right). There was not a clear correlation between the changes we found in our mice and the changes in similar model mice reported previously^25,26^. However, two out of eleven genes whose expressions were changed in the cortex of TDP-43 Tg mice overlapped to those whose splicing was reported to change in previous studies of TDP-43 Tg mice using prion promotor^25^. *Kcnip2* was reported to be one of the genes in which exon skipping was enhanced in the cortex of mutant TDP-43 mice or TDP-43-depleted mice, and it was downregulated in the cortex of our TDP-43 Tg mice (Supplementary Fig. S6). The exon inclusion of *Caly* was enhanced in TDP-43-depleted mice^25^, and the expression of *Caly* was decreased in the cortex in our TDP-43 Tg mice (Supplementary Table S1). *Sort1*, the only gene whose splicing was changed in wild-type TDP-43 Tg mice in the study by Arnold *et al*. was not changed in our TDP-43 Tg mice. Among the genes with changes in expression, *riboflavin kinase* (*Rfk*) was the one for which we observed the largest change in expression in the hippocampus (Fig. 6b, Supplementary Table S1). To confirm the changes in RNA levels observed in the exon array, we performed quantitative RT-PCR for some genes. Changes in the expression of all genes tested including *Rfk and Kcnip2* were confirmed (Supplementary Fig. S6).

### Mild increase in the expression of Riboflavin kinase in the neurons of TDP-43 Tg mice

Among the many gene expression changes we observed in TDP-43 Tg mice, we further focused on riboflavin kinase (Rfk) as it was the one for which we observed the largest change in expression (Fig. 6b, Supplementary Table S1). Rfk is a kinase that phosphorylates riboflavin, and is known to be involved in the production of reactive oxygen species (ROS) as a co-receptor of the TNF receptor^27–29^. The increased expression of *Rfk* in the hippocampus of 8-month-old TDP-43 Tg mice was confirmed by quantitative RT-PCR using SYBR green (Fig. 6c; *p* = 0.0437). To investigate which cell types express *Rfk*, we also performed *in situ* hybridization (Fig. 6d) and immunohistochemical analyses (Fig. 6e,f) with the sections of fresh frozen brains of 8-month-old mice. The expression pattern of Rfk was similar to that of NeuN, indicating that *Rfk* is expressed in all types of neurons (Fig. 6e). We also confirmed the expression of Rfk protein in parvalbumin interneurons (Fig. 6f).

### Rfk can induce neuronal cell death

To investigate whether the increased expression of Rfk can potentially induce neuronal cell death, we overexpressed Rfk in primary hippocampal neurons. We observed increased cell death in neurons expressing Rfk (Fig. 6g), indicating that increased expression of Rfk is sufficient to induce neuronal cell death.

To define the pathway by which interneurons were degenerated in TDP-43 Tg mice, we analysed whether apoptotic cell death was induced in TDP-43 Tg mice. We stained brain sections with anti-cleaved caspase 3 and anti-GAD67 antibodies to detect apoptotic neurons. We did not detect any apoptotic cell death of GABAergic interneurons in both NTg mice and TDP-43 Tg mice (data not shown).

Next, we analysed necroptosis using the RIPK1 antibody. The aggregates were slightly stained with RIPK1 antibody but obvious necroptosis was not detected in TDP-43 Tg mice (Supplementary Fig. S7). Therefore, while inhibitory neurons might have died via necroptosis, there was not a significant increase in cell death of inhibitory neurons in TDP-43 Tg mice.

## Discussion

We found that mice expressing exogenous TDP-43 display memory deficits, and massive p62- and poly-ubiquitin-positive aggregates are observed in the hippocampus of both aged non-Tg mice and TDP-43 Tg mice. We found poly-ubiquitin- and p62-positive aggregates in the parvalbumin neurons had properties similar to polyglucosan bodies known as corpora amylacea^23^ or PAS granules^24^ observed in the brain of aged mice. The number of PAS-stained aggregates was higher in senescence-accelerated mice and upon aging^21^, indicating that aging accelerates the formation of poly-ubiquitin-positive aggregates. Therefore, aging-related cellular changes might be accelerated in TDP-43 Tg mice. We also found that the aggregates consist of calretinin, parvalbumin, p62, poly-ubiquitin, and a small amount of tau protein. These data suggest that aging and upregulation of TDP-43 cause GABAergic interneuron degeneration through an unknown pathway. Moreover, we found that the poly-ubiquitin-positive granules shared the similarity for polyglucosan body known as corpora amylacea that has been shown to be associated with both normal aging and neurodegenerative diseases including Alzheimer’s disease^30^. Our study may provide a possible link of FTD/ALS and other neurodegenerative diseases.

Dysfunction of GABAergic interneurons could lead to hyperactivity of excitatory neurons in the hippocampus, causing impaired memory that is observed in TDP-43 Tg mice. In TDP-43 Tg mice, the number of cleaved caspase 3-positive neurons was slightly increased (Supplementary Fig. S7), but the total number of GABAergic neurons in the hippocampus remained unchanged (Fig. 5d). Therefore, while interneuron cell death may be induced in TDP-43 Tg mice, it did not influence the total number of interneurons. Electrophysiological studies would clarify whether the interneurons in the hippocampus of TDP-43 Tg mice are functionally normal.

Recently, Tg mice expressing mutant TDP-43 were shown to exhibit defects in the inhibitory circuitry due to the decreased activity of parvalbumin-positive GABAergic interneurons^31^. In mutant TDP-43 Tg mice, hyperactivity of somatostatin interneurons leads to the decreased activity of parvalbumin interneurons, resulting in the hyperactivity of pyramidal neurons in layer 5 of the cortex. If excess accumulation of wild-type TDP-43 has an effect similar to or even slightly weaker than that of mutant TDP-43, parvalbumin interneurons might be damaged in the TDP-43 Tg mice we generated as well. If so, we could have detected the defects in interneurons as an increase in the poly-ubiquitin-positive aggregates in the hippocampus of TDP-43 Tg mice.

As opposed to ALS/FTD patients, the cerebellum is affected in TDP-43 mice and accumulations of poly-ubiquitinated aggregates are also detected in the cerebellum (data not shown). This may be due to the overexpression of TDP-43 in the cerebellum, which does not occur in ALS/FTD patients.

We identified several genes misregulated in TDP-43 mice, including genes involved in neuronal function and oxidative stress. Among the genes misregulated in TDP-43 mice, Rfk could induce cell death when upregulated in primary cultures of hippocampal neurons. However, neuronal death was not robustly induced in the hippocampus of TDP-43 Tg mice, so the mechanism underlying how the increase in the aggregates derived from interneurons remains unknown. Another possible pathway to explain the increase in the number of the aggregates is the impaired clearance of poly-ubiquitinated proteins in TDP-43 Tg mice. Hence, poly-ubiquitinated proteins in the aggregates derived from interneurons are bound to p62, signalling that they should be degraded by autophagy. Autophagy is known to reduce with aging. Deficits in autophagic activity are seen in several neurodegenerative diseases beyond FTD/ALS, including Alzheimer’s disease and Huntington’s disease^32,33^. In our exon array analysis, we found Atg10, which is a component of the autophagosome, is downregulated (Supplementary Table S1). Further study is necessary to assess the importance of autophagy in the accumulation of aggregates derived from interneurons in TDP-43 Tg mice. The accumulation of poly-ubiquitin- and p62-positive aggregates that we found in hippocampus of TDP-43 Tg mice could physically interfere with normal neuronal circuitry. Eliminating these aggregates by enhancing autophagy in GABAergic interneurons might mitigate the memory deficits observed in TDP-43 Tg mice.

The FTD/ALS model mice we developed show unique and unreported proteinopathy, which refers to the accumulation of debris of interneurons. The degeneration of interneurons seen in our mouse model could be the very early age-accelerated changes observed in the disease. Moreover, it has been reported that inhibitory interneuron deficits links altered network activity and cognitive dysfunction in models of Alzheimer’s disease^34–36^. Therefore, our TDP-43 Tg mice might be a useful model to study neurological diseases accelerated by aging. The investigation of human postmortem samples will be important for testing if interneuron degeneration occurs in the human brain.

## Methods

### Generation and maintenance of animals

All the experimental protocols with animals were approved by the Animal Care and Use Committee of the RIKEN Brain Science Institute and Nagoya City University, and the animal experiments were performed according to the guidelines of the Ministry of Education, Culture, Sports, Science, and Technology, Japan. FLAG-tagged wild-type human TDP-43 or C-terminal region of human TDP-43 (208–414) were cloned into the exon 2 of the prion protein promoter region in a PrP vector (a gift from Dr. David Borchelt), and injected into pronuclei of fertilized eggs derived from BDF1 mice. The sequences of primers used to genotype the mice are listed in Supplementary Table S2. All the mice were backcrossed with C57BL/6j mice. The mice were housed in a room with a 12 h light/dark cycle, with unrestricted access to food and water. Behavioural tests were performed between 10:00 am and 6:00 pm at the RIKEN Brain Science Institute. Before all the behavioural tests, the mice were housed in a testing room for at least 30 min to allow acclimatisation to the testing environment. After the test, the testing apparatus was cleaned with 70% ethanol to prevent a bias due to olfactory cues.

### Y-maze test

The Y-maze test was performed using the 3-arm Y-maze system with an attached camera and computer (model YM-3002, O’HARA & CO. LTD, Tokyo, Japan) under low light conditions (20 Lux). Mice began a single trial at the end of one arm and were allowed to explore the Y-maze freely. The movement of mice was analysed using the Time YM1 software (O’HARA & CO. LTD).

### Rotarod test

The motor function of each mouse was tested with a rotarod device (MK-610A, Muromachi Kikai). The rotarod initially ran at 3 rpm and accelerated to 30 rpm over 300 seconds, increasing by 1 rpm every 10 seconds. Each mouse underwent three sessions with a 1-hour inter-session interval.

### Contextual and cued fear conditioning test

The fear conditioning test was performed using the fear conditioning test system CL-1020 (O’HARA & CO. LTD). Mice were placed in a chamber and provided with a conditioned stimulus (CS) of 65 dB noises and an unconditioned stimulus (US) in the form of a foot shock (0.15 mA) twice (conditioning). On the following day, the mice were returned to the conditioning chamber and allowed to explore the chamber without either the CS or the US (contextual fear test). Mice were then placed in another chamber with different properties from the conditioning chamber, and the CS was presented (cued fear test).

### Immunohistochemistry

Anesthetized mice were intracardially perfused with 4% paraformaldehyde in phosphate buffer, and the brains and spinal cord were post-fixed overnight at 4 °C in the same buffer, and embedded in paraffin. The brain sections (6 μm) were deparaffinised, autoclaved at 120 °C for 2 min and blocked with 3% donkey serum and 1% bovine serum albumin in Tris-buffered saline containing 0.1% TritonX-100. The brain sections were then probed with the following primary antibodies: FLAG (Sigma-Aldrich, M2, ×500), TDP-43 (ProteinTech, #10782-2-AP, ×500), ChAT (Millipore, #AB144P, ×100), IbaI (Wako, #019–19741, ×500), GFAP (Sigma, #G3893), Myelin Basic Protein (Millipore, #AB980), NeuN (Millipore, #MAB377), GAD-67 (Millipore, #MAB5406, ×500), Parvalbumin (Millipore, #MAB1572, ×1000), Calretinin (Millipore, #MAB1568, ×1000), p62 (MBL, #PM045, ×500), multi-Ubiquitin (MBL, #D058-3, ×300), MAP2 (Abcam, #AB7756, ×600), Tau1 (Millipore, #MAB3420, ×200), Rfk (Abgent, #AP7183a, ×50), RIPK-1 (Novus Biogenesis, #NBP1-77077, ×400), and Cleaved Caspase-3 (Cell Signalling, #9661 S, ×400); and with the following secondary antibodies: fluorescently conjugated anti-rabbit, anti-rat, anti-mouse, and anti-goat antibodies (Alexa Flour, Life Technologies). For the DAB staining, the sections were blocked using the avidin-biotin blocking kit (Vector Lab, #SP-2001), probed with primary antibodies and biotinylated secondary antibodies. The staining was visualised by the avidin-biotin complex immunoperoxidase technique using the VECSTATIN Elite ABC system (Vector Lab). The images were obtained using a Zeiss LSM 5 exciter confocal microscope (Carl Zeiss) and the Fluorescence Microscope system BioVio BZ-9000 (KEYENCE, Japan)

### Western blotting

The tissue was homogenized with the RIPA buffer (10 mM Tris-HCl pH 7.3, 150 mM NaCl, 1% TritonX-100, 0.1% SDS, and protease inhibitor cocktail (Roche) and phosphatase inhibitor (PhosSTOP, Roche) on ice with sonication. After centrifuging the homogenate at 15,000 rpm for 10 min, the soluble fraction was collected and the concentration of protein was quantified by the BCA protein assay kit (Bio-Rad). Either 10 or 20 μg of the protein sample was used for the SDS-PAGE. The following primary antibodies were used for immunoblotting: FLAG (Sigma-Aldrich, M2), TDP-43 (ProteinTech), GAPDH (Millipore, #MAB374), β-actin (Sigma, #A5441).

### Electron microscopy

Conventional electron microscopy and Correlative light- and immunoelectron microscopy (CLEM) on Tokuyasu cryosections were performed as described previously^37,38^ with some modifications. Briefly, C57BL/6 mice at 25 months were deeply anesthetized with pentobarbital (25 mg/kg i.p.) and fixed by cardiac perfusion either with 2% paraformaldehyde (PA) and 2% glutaraldehyde (GA) or 4% PA buffered with 0.1 mol/L phosphate buffer (PB; pH7.2) for conventional electron microscopy and immunoelectron microscopy, respectively. Brain tissues were further immersed in the same fixative overnight at 4 °C, and 1-mm thick sagittal slices of hippocampal tissue were prepared. For conventional microscopy, samples were postfixed with 2% OsO4 in 0.1 mol/L PB (pH7.2), block-stained in 1% aqueous uranyl acetate, dehydrated with a graded series of alcohol, and embedded in Epon 812 (TAAB, Reading, UK). Ultrathin sections were cut with a Leica UC6 ultramicrotome (Leica Microsystems, Vienna, Austria), stained with uranyl acetate and lead citrate, and observed with a Hitachi HT7700 electron microscope (Hitachi, Tokyo, Japan). For immunoelectron microscopy, samples were washed thoroughly with 7.5% sucrose in 0.1 M PB (pH7.2), embedded in 12% gelatin in 0.1 M PB (pH7.2), rotated in 2.3 M sucrose in 0.1 M PB (pH7.2) overnight at 4 °C, placed on a specimen holder (Leica Microsystems, Vienna, Austria), and quickly plunged into liquid nitrogen until used. Approximately 400 nm thick sections were cut at –80 °C with a Leica UC7/FC7. Thereafter, sections were incubated for 1 hour at room temperature (RT) each in rabbit anti-p62 (1:20, Wako Pure Chemical Industries, Osaka, Japan) primary antibody, and Alexa 488 conjugated donkey anti-rabbit IgG (1:200, Invitrogen Life Technologies, Carlsbad, CA). Following counter staining with DAPI, sections were coverslipped with 50% glycerol in distilled water and observed by a BZ-X700 microscope (Keyence, Osaka, Japan). After fluorescent microscopic identification of punctate p62-positive structures in the hippocampus, ultrathin cryosections (~70-nm) were cut with a Leica UC7/FC7 at about –120 °C, picked up with a 1:1 mixture of 2% methylcellulose and 2.3 M sucrose, and transferred to a nickel finder grid for subsequent electron microscopy observations. Sections were rinsed with PBS containing 0.02 M glycine, treated with 1% BSA in PBS, and incubated overnight at 4 °C with rabbit anti-p62 (1:20), following the sequential incubations for 1 hour at RT with Alexa488 conjugated donkey anti-rabbit IgG (1:200) and protein A gold with 10 nm colloidal gold particles, respectively (1:50, Cell Microscopy Center, University Medical Center Utrecht, Utrecht, the Netherlands). The sections were then fixed with 1% GA in PBS. Grids were independently coverslipped with 50% glycerol in distilled water and observed by BZ-X700 microscope. The coverslips were unmounted from the object slide in a 10 cm dish with distilled water. The grids were embedded in a thin layer of 2% methylcellulose with 0.4% uranyl acetate (pH 4.0), air-dried, and then ultrastructures corresponding to those with coarse granular fluorescence were observed with a Hitachi HT7700 electron microscope For the control experiments, ultrathin sections were reacted only with the gold particle- and/or Alexa 488 conjugated secondary antibody.

### Exon array and qRT-PCR

The total RNA was extracted using the mirVANA miRNA isolation kit (Ambion) according to the manufacturer’s instructions, and its integrity was assessed using a Bioanalyzer (Agilent). The samples were prepared using the WT Expression Kit (Ambion) and the GeneChip Terminal Labelling Kit (Affymetrix) according to the manufacturer’s instructions, and were hybridised to the Mouse Exon 1.0 ST Array (Affymetrix). The data was analysed using GeneSpring GX (Agilent). The detailed method used for quantitative RT-PCR has been previously described elsewhere^39^. Briefly, it was performed using the SYBR green mix (ABI) and gene-specific primers using the Thermal Cycler Dice Real Time System II (Takara Bio). More than three biological replicates per group and three technical replicates were applied. The relative mRNA expression was calculated using the standard curve method by normalizing the absolute expression level to that of ß-actin and relative to that of the control samples. The sequences of primers designed using the Primer3 software are available in Supplementary Table S2.

### *In situ* hybridisation

*In situ hybridisation* (ISH) was carried out as previously described^40^. Briefly, mice were perfused with 4% PFA in phosphate buffer using DEPC-treated water, and the brain tissue was dissected and post-fixed in the same buffer overnight. The tissue was frozen on the next day, and brain sections (20 μm) were prepared within a week. The sections were treated with 0.1 N HCl and Protease K, fixed, and acetylated. After pre-hybridisation in 50% formamide, 2x SSC, 1x Denhardt’s, 10 mM EDTA, 50 mg/ml tRNA, and 0.01% Tween20 at 55 °C for 1-2 h, the sections were hybridised with digoxigenin (DIG)-labelled RNA probes prepared using the DIG RNA labelling kit (Roche, #11175025910) in pre-hybridisation buffer supplemented with 5% dextran sulphate at 55 °C overnight. After RNaseH treatment to digest the unhybridised DIG-RNA, the brain sections were intensively washed with a low ionic buffer, and incubated with AP-conjugated anti-DIG antibody (Roche). The signal was visualised with NBT-BCIP. A fragment (1083–2036 nt) of the mouse Rfk gene (NM_019437) was cloned into pBluescript II and used as the template for probe synthesis.

### Cell death assay

The hippocampus was dissected from E18 ICR mice and dissociated with trypsin-EDTA. The cells were cultured on poly-L-lysine-coated cover glass in Neurobasal medium supplemented with 2% B27, 5% FBS, and Penicillin-Streptomycin. The primary cultured hippocampal neurons were transfected with GFP or GFP-Rfk at DIV (days *in vitro*) 3, and the cells were fixed and co-stained with anti-GFP and anti-cleaved caspase-3 (CC3) antibodies to assess cell death at DIV 6, 8, and 10. The number of CC3-positive cells divided by the number of GFP-positive was calculated.

### Statistical analysis

The unpaired t test, unpaired t test with Welch’s correction, and Mann-Whitney U test were used. The data except Fig. 5d are presented as mean ± SEM. The data in Fig. 5d is presented as box and whiskers (min to max). Significance level thresholds of *p < 0.05, **p < 0.01, and ***p < 0.001 were used. The statistical analysis was performed using Excel and GraphPad Prism 6 (GraphPad Software Inc.). Confocal images were prepared with Adobe Photoshop CS4.

## Electronic supplementary material

Supplementary Information

Supplementary Movie S1
