# Supplementary material for: TDP-43 accelerates age-dependent degeneration of interneurons
Source: Sci Rep. 2017 Nov 2;7:14972. doi: 10.1038/s41598-017-14966-w (PMC5668320; doi:10.1038/s41598-017-14966-w)
Supplement: Supplementary file 1 — Supplementary Information [file 41598_2017_14966_MOESM1_ESM.pdf]

TDP-43 accelerates age-dependent degeneration of interneurons

Hitomi Tsuiji[1,2], Ikuyo Inoue[2], Mari Takeuchi[1], Asako Furuya[2], Yuko Yamakage[1], Seiji Watanabe[2,3], Masato Koike[4], Mitsuharu Hattori[1], Koji Yamanaka[2,3*]*

[1]*Department of Biomedical Science, Graduate School of Pharmaceutical Sciences, Nagoya City University, Nagoya, Aichi 467-8603, Japan*

[2]*Laboratory for Motor Neuron Disease, RIKEN Brain Science Institute, Wako, Saitama 351-0198, Japan*

[3]*Department of Neuroscience and Pathobiology, Research Institute of Environmental Medicine, Nagoya University, Nagoya, Aichi 464-8601, Japan*

[4]*Department of Cell Biology and Neuroscience, Juntendo University Graduate School of Medicine, Bunkyo-ku, Tokyo 113-8421, Japan.*

*Corresponding authors:

Hitomi Tsuiji

3-1 Tanabe-dori, Mizuho-ku, Nagoya, Aichi 467-8603, Japan.

Tel: +81-52-836-3789; Fax: +81-52-836-3756

E-mail: hitomitsuiji@phar.nagoya-cu.ac.jp

Koji Yamanaka

Furo-cho, Chikusa-ku, Nagoya, Aichi 464-8601, Japan

Tel: +81-52-789-3865; Fax: +81-52-789-3891

E-mail: kojiyama@riem.nagoya-u.ac.jp

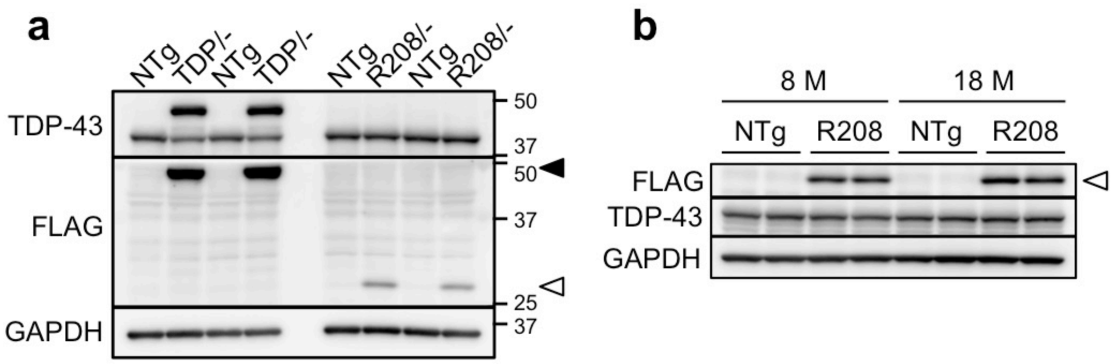

Supplementary Figure S1. Immunoblots of brain tissue of non-Tg (NTg), heterozygous TDP-43 Tg (TDP), and heterozygous TDP-43 Tg (R208) mice.

Whole brain tissues of mice at 8 months (**a** and **b**) and 18 months (**b**) of age with the indicated genotypes were immunoblotted with anti-TDP-43, anti-FLAG, or anti-GAPDH antibodies. The filled and open arrowheads denote full-length hTDP-43-FLAG and R208-FLAG, respectively.

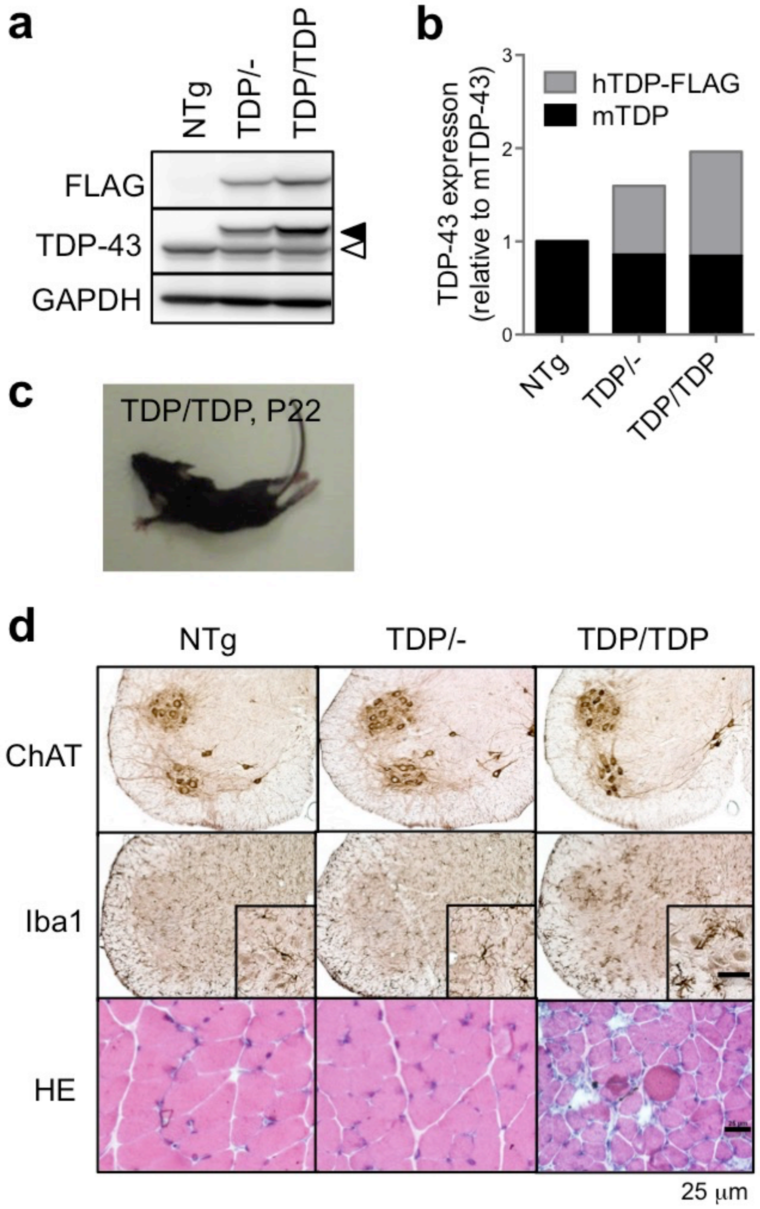

Supplementary Figure S2. Severe paralysis of homozygous TDP-43 Tg mice.

(**a**) Immunoblots of the brain tissue of non-Tg, heterozygous (TDP/-) and homozygous (TDP/TDP) Tg mice at P22. (**b**) Quantitative analysis of the amount of TDP-43 relative to wild-type TDP-43 in non-Tg mice; n=2, each. (**c**) Severe hindlimb paralysis of homozygous TDP-43 Tg mice at P22 is shown. (**d**) Immunohistochemistry and HE staining of the spinal cord and skeletal muscle of TDP-43 Tg mice. Motor neurons remain intact, but microgliosis and atrophic and degenerated muscle fibres were observed in the lumbar spinal cord and gastrocnemius muscle of P22 homozygous TDP-43 Tg mice (TDP/TDP); Bars: 25 μm.

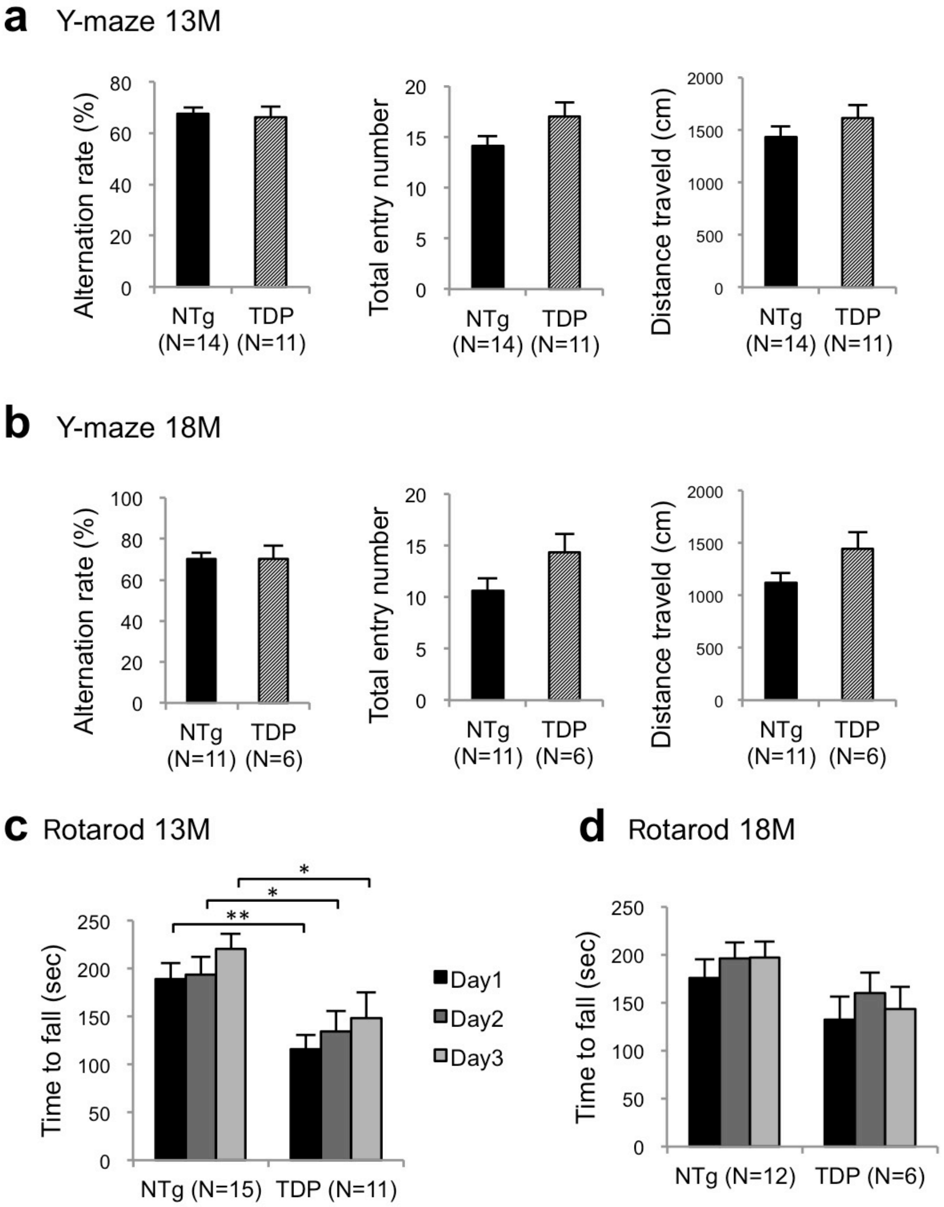

Supplementary Figure S3. Mild impairment in motor learning in heterozygous TDP-43 Tg mice.

(**a, b**) Quantitative analysis of the Y-maze test data. The mean alteration rate, total number of entries, and total distance travelled of non-Tg (NTg) and TDP-43 Tg (TDP) mice at 13 months (**a**) and 18 months (**b**) of age are plotted. (**c, d**) Quantitative analysis of the rotarod test data. The mean holding times on the rotating rod at 13 months (**c**) and 18 months (**d**) of age over three sequential trials are shown. The data are presented as mean ± SEM. Unpaired t-test, $*p < 0.05$, $**p < 0.01$.

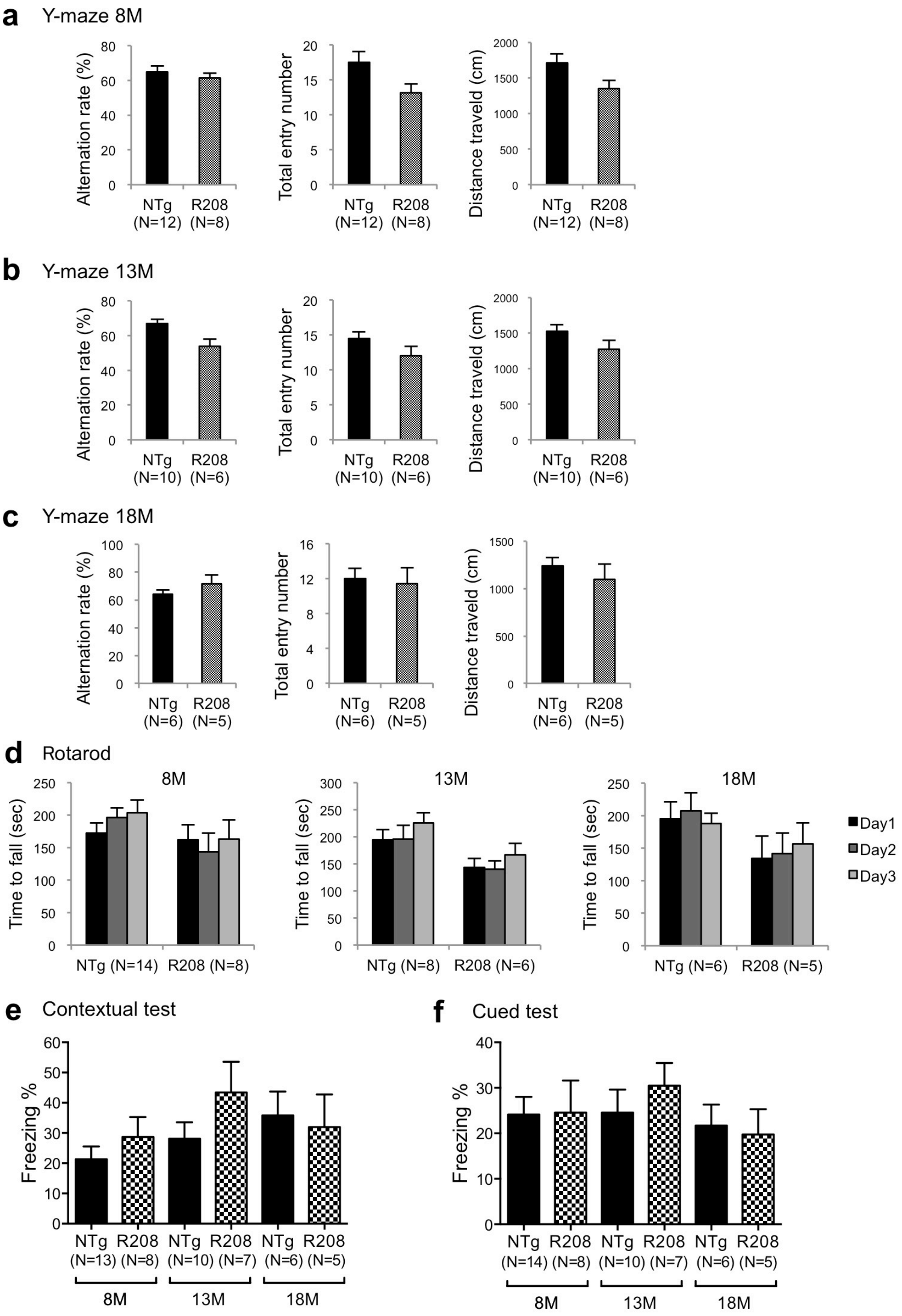
a
Y-maze 8M
Alternation rate (%)
Total entry number
Distance traveld (cm)
NTg (N=12)
R208 (N=8)
b
Y-maze 13M
NTg (N=10)
R208 (N=6)
c
Y-maze 18M
NTg (N=6)
R208 (N=5)
d
Rotarod
8M
13M
18M
Time to fall (sec)
NTg (N=14)
R208 (N=8)
NTg (N=8)
R208 (N=6)
NTg (N=6)
R208 (N=5)
Day1
Day2
Day3
e
Contextual test
Freezing %
NTg (N=13)
R208 (N=8)
NTg (N=10)
R208 (N=7)
NTg (N=6)
R208 (N=5)
8M
13M
18M
f
Cued test
Freezing %
NTg (N=14)
R208 (N=8)
NTg (N=10)
R208 (N=7)
NTg (N=6)
R208 (N=5)
8M
13M
18M

Supplementary Figure S4. No significant impairment in motor learning and memory in heterozygous R208 Tg mice.

(**a-c**) Quantitative analysis of the Y-maze test data. The mean alteration rate, total number of entries, and total distance travelled of non-Tg (NTg) and R208 Tg (R208) mice at 8 months (**a**), 13 months (**b**), and 18 months (**c**) of age are plotted. (**d**) Quantitative analysis of the rotarod test data. The mean holding times on the rotating rod at 8 months (left), 13 months (middle), and 18 months (right) of age over three sequential trials are shown. (**e, f**) Quantitative analysis of the contextual and cued fear conditioning test data. The percentage of mean freezing times in the contextual test (**e**) and cued test (**f**) at the indicated ages is plotted.

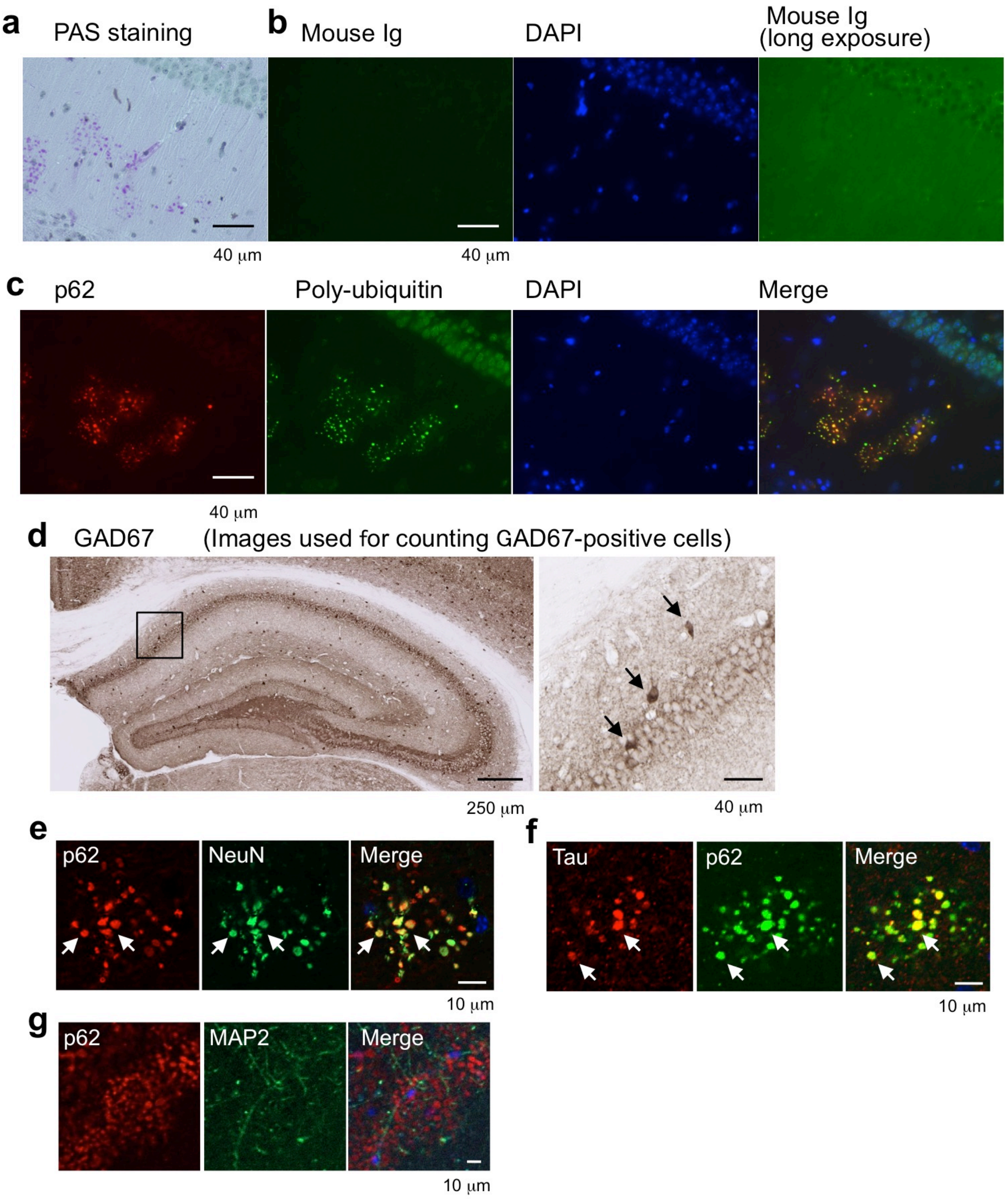

Supplementary Figure S5. Massive poly-ubiquitin- and p62-positive aggregates derived from GABAergic interneurons in the hippocampus of aged mice.

(**a-c**) Staining of continuous sections (6 μm) of the hippocampus of TDP-43 Tg mice. PAS staining (**a**), immunostaining with mouse Ig as a negative control (**b**), and immunostaining with anti-poly-ubiquitin and anti-p62 antibodies (**c**). (**d**) Immunostaining of the hippocampus of TDP-43 Tg mice with anti-GAD-67 antibody. The magnified image in the boxed region of the left panel is shown (right). The arrows

denote GAD-67-positive cells. (**e-g**) Immunofluorescence stainings of aggregates in the hippocampus of aged mice with p62 and anti-NeuN antibodies (**e**), anti-p62 and anti-Tau (Tau1) antibodies (**f**), and anti-p62 and anti-MAP2 antibodies (**g**).

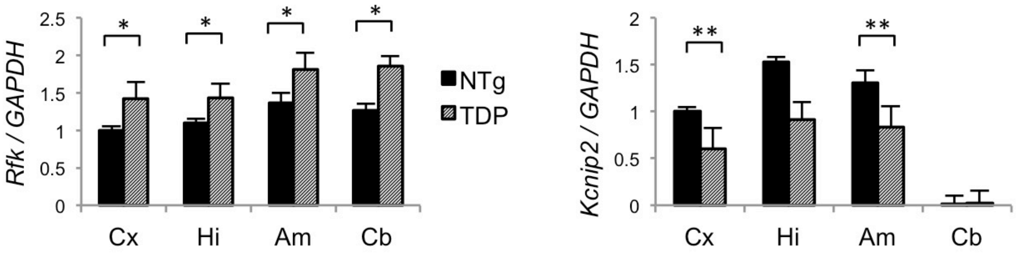

Supplementary Figure S6. Changes in the expression of *Rfk* and *Kcnip2* in TDP-43 Tg mice.

Quantitative PCR analysis of the *Rfk* (left) and *Kcnip2* (right) mRNAs. Unpaired t-test, *$p < 0.05$, **$p < 0.01$, n=3 each.

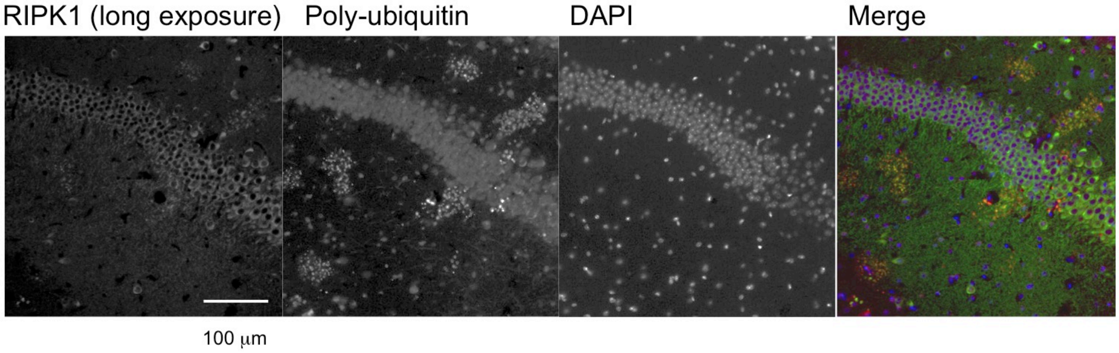

Supplementary Figure S7. Immunofluorescence staining of the hippocampus of aged TDP-43 Tg mice with anti-multi-ubiquitin and anti-RIPK1 antibodies.

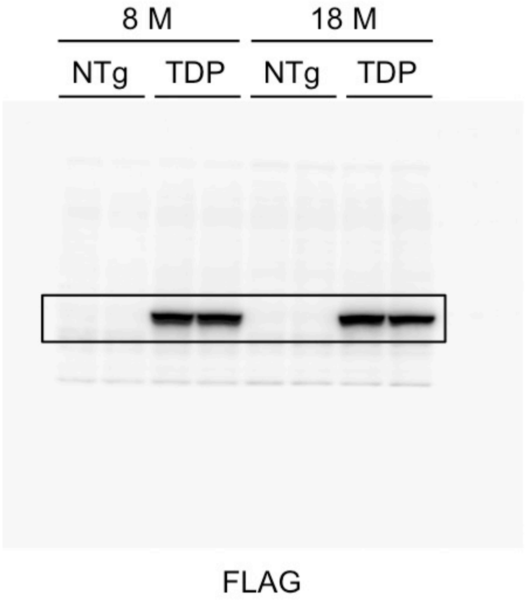

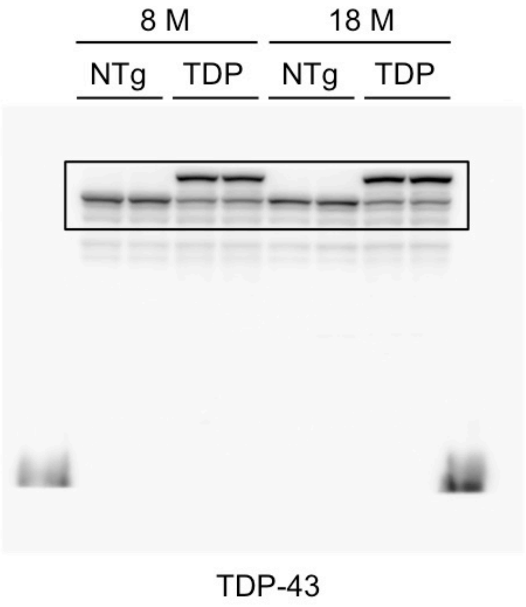

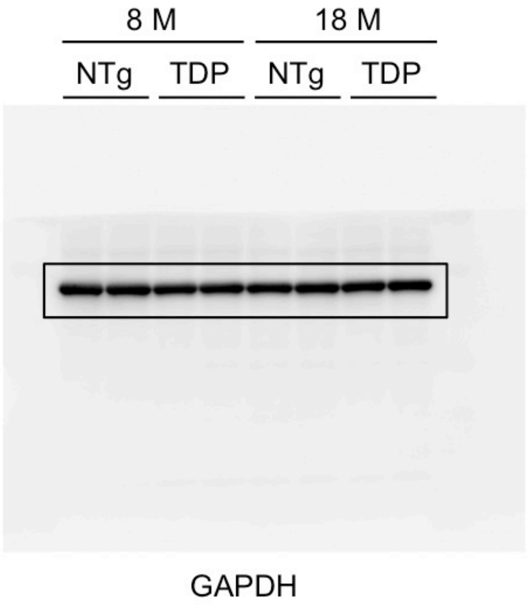

Supplementary Figure S8. Original Western blots for Figure 1e.

Full length Western blots of brain tissue lysates from the indicated mice presented in Figure 1e. Rectangles refer to the regions that were used to generate a figure panel.

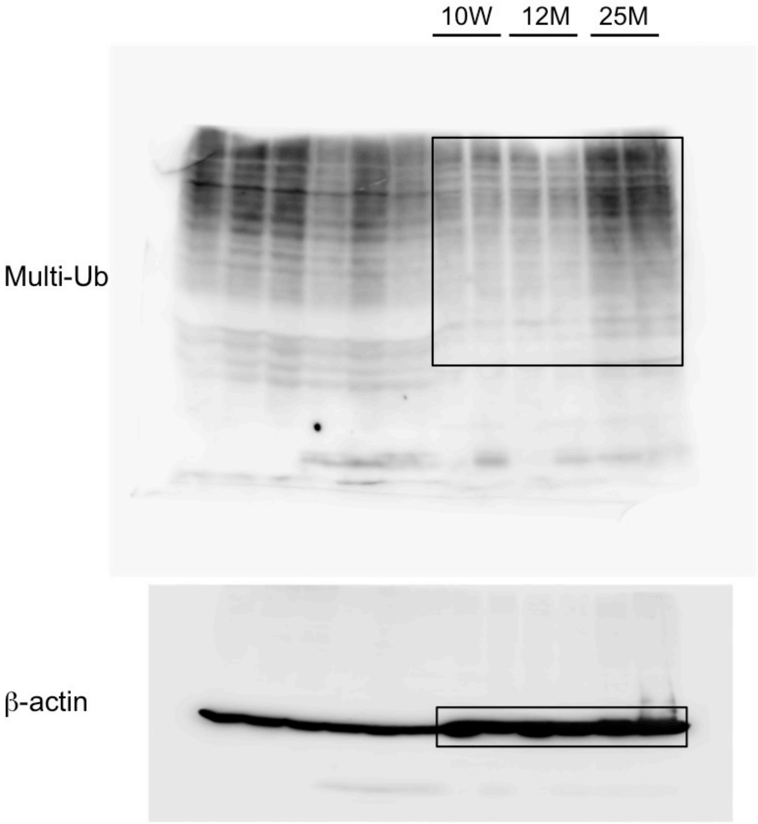

Supplementary Figure S9. Original Western blots for Figure 5c.

Full length Western blots of brain tissue lysates from the indicated mice presented in Figure 5c. Rectangles refer to the regions that were used to generate a figure panel.

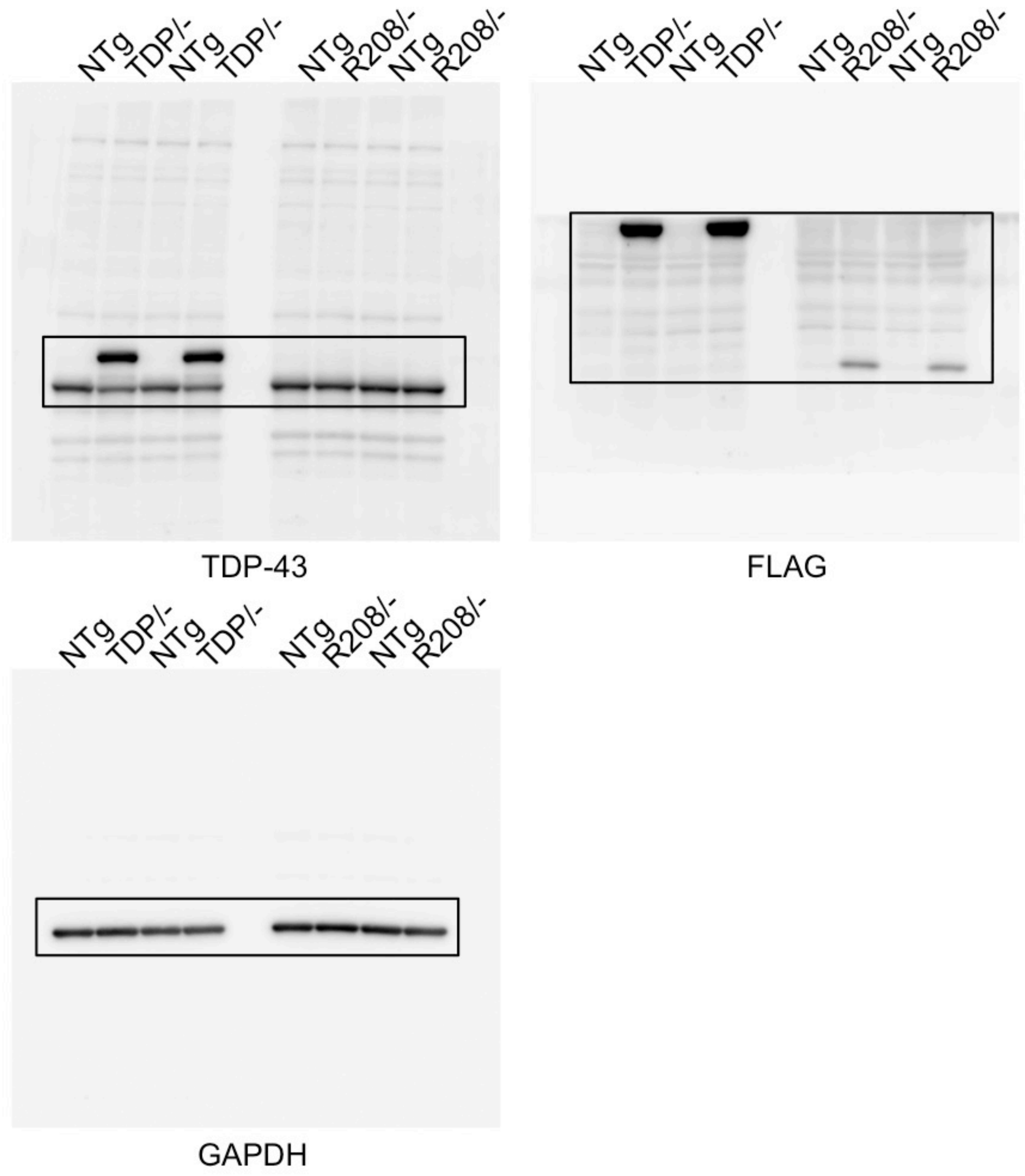

Supplementary Figure S10. Original Western blots for Supplementary Figure S1a. Full length Western blots of brain tissue lysates from the indicated mice presented in Supplementary Figure S1a. Rectangles refer to the regions that were used to generate a figure panel.

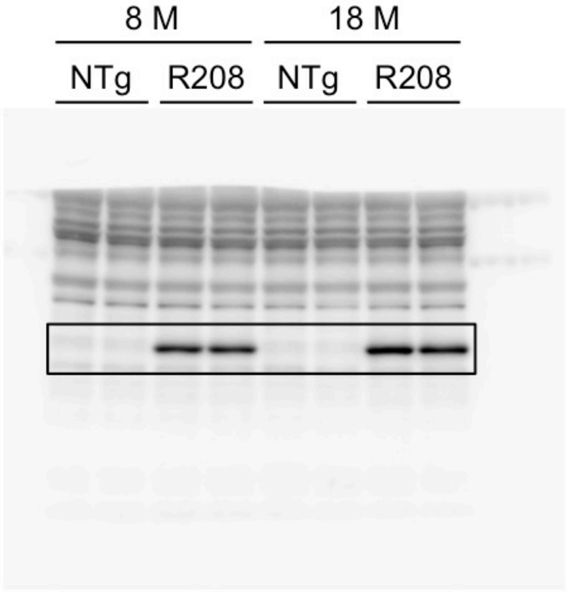

FLAG

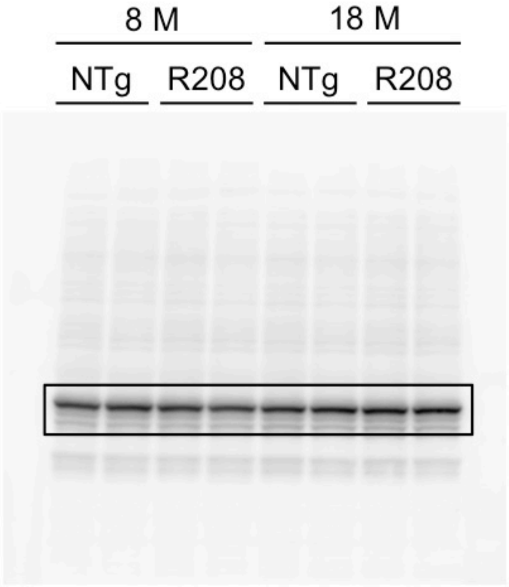

TDP-43

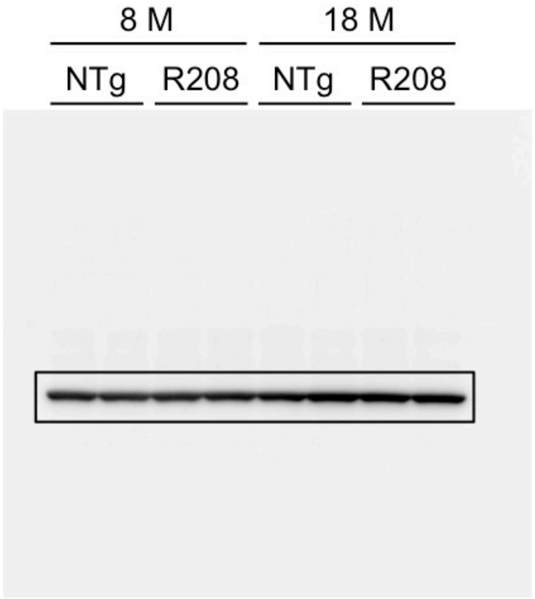

GAPDH

Supplementary Figure S11. Original Western blots for Supplementary Figure S1b. Full length Western blots of brain tissue lysates from the indicated mice presented in Supplementary Figure S1b. Rectangles refer to the regions that were used to generate a figure panel.

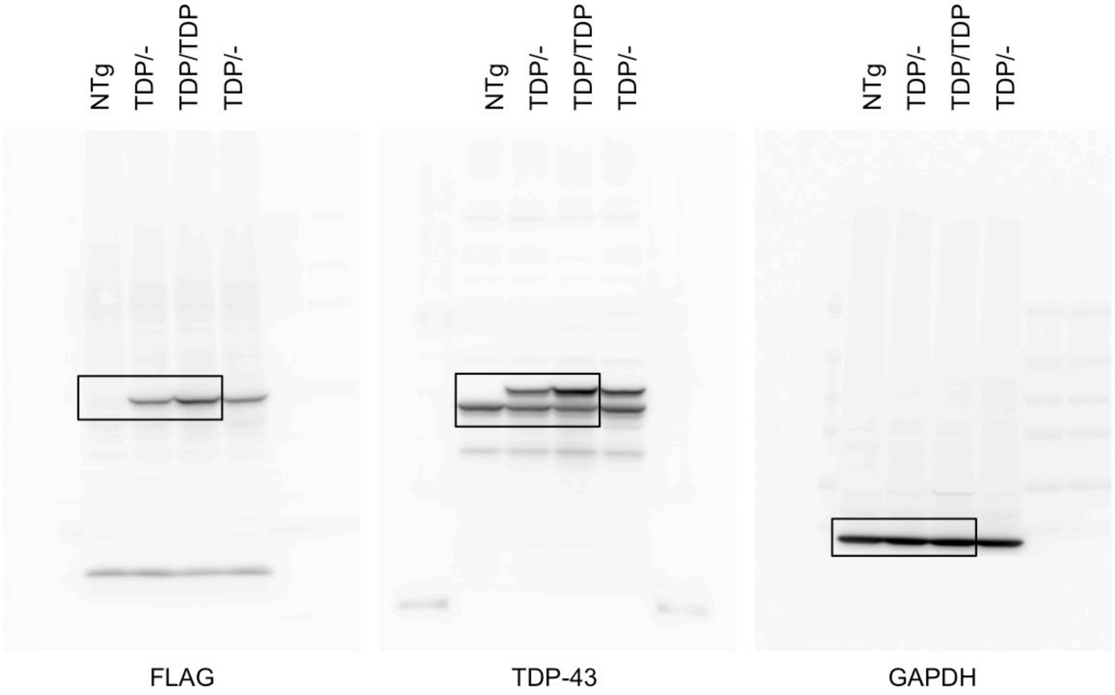

Supplementary Figure S12. Original Western blots for Supplementary Figure S2a. Full length Western blots of brain tissue lysates from the indicated mice presented in Supplementary Figure S2a. Rectangles refer to the regions that were used to generate a figure panel.

Supplementary Movie S1. Homozygous TDP-43 Tg mice at P20.

Supplementary Table S1: Genes differentially expressed between heterozygous TDP-43 Tg mice and NTg mice (p < 0.05, fold change > 1.2)

Cortex (Tg vs nTg)

Transcripts Cluster Id	Fold Change	p value	Genesymbol	Genedescription
6757331	-1.32	0.0091	Kcnip2	Kv channel-interacting protein 2
6837006	-1.28	0.0479	Tardbp	TAR DNA binding protein
6854487	-1.27	0.0376	Olfr665	olfactory receptor 665
6868386	-1.25	0.0066	Chid1	chitinase domain containing 1
6868404	-1.25	0.0112	Caly	calcyon neuron-specific vesicular protein
6873368	1.22	0.0302	Lyrm2	LYR motif containing 2
6912504	1.30	0.0450	Pvalb	parvalbumin
6926916	1.33	0.0014	Eif3m	eukaryotic translation initiation factor 3, subunit M
6963165	1.34	0.0475	Prune2	prune homolog 2 (Drosophila)
6972114	1.48	0.0073	Dusp1	dual specificity phosphatase 1
6972227	1.59	0.0012	Rfk	riboflavin kinase

Hippocampus (Tg vs nTg)

Transcripts Cluster Id	Fold Change	p value	Genesymbol	Genedescription
6781396	-1.32	0.0273	Med9	mediator of RNA polymerase II transcription, subunit 9 homolog (yeast)
6788411	-1.30	0.0431	Atox1	ATX1 (antioxidant protein 1) homolog 1 (yeast)
6780715	-1.30	0.0145	Olfr1394	olfactory receptor 1394
6879989	-1.30	0.0495	Olfr1279	olfactory receptor 1279
6845028	-1.25	0.0200	Gp5	glycoprotein 5 (platelet)
6945788	-1.25	0.0064	Tas2r143	taste receptor, type 2, member 143
6933476	-1.24	0.0181	Ttc28	tetratricopeptide repeat domain 28
6945814	-1.23	0.0103	Olfr450	olfactory receptor 450
6956581	-1.23	0.0455	Ghrl	ghrelin
6971415	-1.22	0.0452	Pycard	PYD and CARD domain containing
6983820	-1.22	0.0237	Rln3	relaxin 3

6810255	-1.21	0.0267	Ankrd55	ankyrin repeat domain 55
6773235	1.20	0.0151	Amd1|Amd2	S-adenosylmethionine decarboxylase 1 | S-adenosylmethionine decarboxylase 2
6962495	1.20	0.0117	Crebzf	CREB/ATF bZIP transcription factor
6802349	1.20	0.0012	Npc2	Niemann Pick type C2
6990859	1.21	0.0267	Irak1bp1	interleukin-1 receptor-associated kinase 1 binding protein 1
6783784	1.21	0.0056	Abi3	ABI gene family, member 3
6747478	1.22	0.0194	3110035E14Rik	RIKEN cDNA 3110035E14 gene
6829647	1.22	0.0051	Mtdh	metadherin
6933306	1.22	0.0262	Dr1	down-regulator of transcription 1
6906267	1.22	0.0264	Glrb	glycine receptor, beta subunit
6756541	1.22	0.0207	Cd34	CD34 antigen
6969814	1.22	0.0487	D630004N19Rik	RIKEN cDNA D630004N19 gene
6783255	1.22	0.0430	Rnft1	ring finger protein, transmembrane 1
6920290	1.23	0.0074	Manea	mannosidase, endo-alpha
6996297	1.23	0.0470	Rab11a	RAB11a, member RAS oncogene family
6762784	1.24	0.0369	Rgs2	regulator of G-protein signaling 2
6799836	1.24	0.0112	Dld	dihydrolipoamide dehydrogenase
6786561	1.24	0.0352	Ugp2	UDP-glucose pyrophosphorylase 2
6790508	1.24	0.0242	Tmem49|Mir21	transmembrane protein 49 | microRNA 21
6953903	1.25	0.0137	Kbtbd2	kelch repeat and BTB (POZ) domain containing 2
6964053	1.25	0.0058	Rbbp6	retinoblastoma binding protein 6
6959557	1.25	0.0333	Zfp260	zinc finger protein 260
6788617	1.25	0.0217	Olfr323	olfactory receptor 323
6981924	1.26	0.0174	Asah1	N-acylsphingosine amidohydrolase 1
6913344	1.27	0.0057	5730528L13Rik	RIKEN cDNA 5730528L13 gene
6920276	1.28	0.0314	Fut9	fucosyltransferase 9
6970442	1.30	0.0463	Rnf141	ring finger protein 141
6868404	1.58	0.0028	Rfk	riboflavin kinase

Amygdala Tg vs nTg

Transcripts Cluster Id	Fold Change	p value	Genesymbol	Genedescription
6873368	-1.50	0.0481	Kcnip2	Kv channel-interacting protein 2
6876072	-1.35	0.0199	Cstad	CSA-conditional, T cell activation-dependent protein
6957763	-1.31	0.0183	Arhgdib	Rho, GDP dissociation inhibitor (GDI) beta
6800890	-1.29	0.0421	Eapp	E2F-associated phosphoprotein
6812444	-1.28	0.0031	Ssr1	signal sequence receptor, alpha
6769617	-1.25	0.0127	Ccdc53	coiled-coil domain containing 53
6942672	-1.24	0.0316	Cox19	COX19 cytochrome c oxidase assembly homolog (S. cerevisiae)
6875375	-1.24	0.0411	Commd3	COMM domain containing 3
6934061	-1.24	0.0451	Tmem116	transmembrane protein 116
6839932	-1.23	0.0195	Ap2m1	adaptor protein complex AP-2, mu1
6774400	-1.21	0.0399	Srgn	serglycin
6752409	-1.20	0.0493	Mki67ip	Mki67 (FHA domain) interacting nucleolar phosphoprotein
6854579	1.20	0.0221	C230013L11Rik	RIKEN cDNA C230013L11 gene
6977048	1.22	0.0243	A230052G05Rik	RIKEN cDNA A230052G05 gene
6900082	1.22	0.0493	Tspan2	tetraspanin 2
6833140	1.29	0.0168	B430209F14Rik	RIKEN cDNA B430209F14 gene
6841969	1.31	0.0116	Fam60a	family with sequence similarity 60, member A
6889720	1.32	0.0171	Olfr1295	olfactory receptor 1295
6860035	1.32	0.0276	Pnet-ps	prenatal ethanol induced mRNA, pseudogene
6868404	1.47	0.0042	Rfk	riboflavin kinase

Cerebellum (Tg vs nTg)

Transcripts Cluster Id	Fold Change	p value	Genesymbol	Genedescription
6780747	-1.66	0.0325	Olfr1381|Olfr1373|Olfr1371|Olfr1382|Olfr1380	olfactory receptor 1381 | olfactory receptor 1373 | olfactory receptor 1371 | olfactory receptor 1382 | olfactory receptor 1380
6789652	-1.55	0.0406	Olfr411	olfactory receptor 411

6850063	-1.47	0.0361	Fkbpl	FK506 binding protein-like
6868183	-1.42	0.0127	Olfr1442	olfactory receptor 1442
6811519	-1.41	0.0422	Hist1h4k	histone cluster 1, H4k
7014142	-1.36	0.0033	Frmpd3	FERM and PDZ domain containing 3
6980107	-1.35	0.0417	Cd209c	CD209c antigen
6836959	-1.34	0.0077	Apol7c	apolipoprotein L 7c
6900352	-1.34	0.0464	Romo1	reactive oxygen species modulator 1
6900141	-1.33	0.0345	Ptpn22	protein tyrosine phosphatase, non-receptor type 22 (lymphoid)
6981091	-1.31	0.0107	1810011O10Rik	RIKEN cDNA 1810011O10 gene
6805404	-1.31	0.0109	Hist1h2aa	histone cluster 1, H2aa
6836949	-1.30	0.0344	Apol9a	apolipoprotein L 9a
6972292	-1.30	0.0216	6330512M04Rik	RIKEN cDNA 6330512M04 gene
6880476	-1.29	0.0323	Chst14	carbohydrate (N-acetylgalactosamine 4-0) sulfotransferase 14
7007728	-1.29	0.0017	B230220N19Rik	RIKEN cDNA B230220N19 gene
6892056	-1.28	0.0404	Nanp	N-acetylneuraminic acid phosphatase
6983980	-1.27	0.0329	F830004M19Rik	RIKEN cDNA F830004M19 gene
6988772	-1.27	0.0249	Apoa5	apolipoprotein A-V
6966343	-1.27	0.0355	Fxyd3	FXYD domain-containing ion transport regulator 3
6763247	-1.25	0.0295	Fam163a	family with sequence similarity 163, member A
6992920	-1.25	0.0389	Rpsa	ribosomal protein SA
6760368	-1.24	0.0309	Nmur1	neuromedin U receptor 1
6966325	-1.24	0.0430	Hamp2	hepcidin antimicrobial peptide 2
6972742	-1.23	0.0458	Olfr5	olfactory receptor 5
6983320	-1.22	0.0211	Hmgn2	high mobility group nucleosomal binding domain 2
6840527	-1.22	0.0240	1500031L02Rik	RIKEN cDNA 1500031L02 gene
6876211	-1.22	0.0495	Ak1	adenylate kinase 1
6972227	-1.21	0.0426	Chid1	chitinase domain containing 1
6890415	-1.21	0.0199	Mageb3|Mageb1|Mageb2	melanoma antigen, family B, 3 | melanoma antigen, family B, 1 | melanoma antigen, family B, 2

6958897	-1.20	0.0121	Ceacam9|Ceacam15	carcinoembryonic antigen-related cell adhesion molecule 9 | carcinoembryonic antigen-related cell adhesion molecule 15
6929676	-1.20	0.0150	Trim54	tripartite motif-containing 54
7006853	-1.20	0.0398	Naip1|Naip2	NLR family, apoptosis inhibitory protein 1 | NLR family, apoptosis inhibitory protein 2
6915037	-1.20	0.0062	Dimt1	DIM1 dimethyladenosine transferase 1-like (S. cerevisiae)
6961932	-1.20	0.0489	5730590G19Rik	RIKEN cDNA 5730590G19 gene
6959127	1.20	0.0065	Cadm4	cell adhesion molecule 4
6918098	1.20	0.0004	Rcc2	regulator of chromosome condensation 2
6841097	1.21	0.0427	Naa50	N(alpha)-acetyltransferase 50, NatE catalytic subunit
6966869	1.21	0.0233	2310044H10Rik	RIKEN cDNA 2310044H10 gene
7015995	1.22	0.0430	Zfp300	zinc finger protein 300
6980968	1.22	0.0358	Ap3m2	adaptor-related protein complex 3, mu 2 subunit
6852068	1.22	0.0391	Clip4	CAP-GLY domain containing linker protein family, member 4
6774311	1.22	0.0399	Unc5b	unc-5 homolog B (C. elegans)
6801324	1.22	0.0119	Klhl28	kelch-like 28 (Drosophila)
6908461	1.22	0.0289	S1pr1	sphingosine-1-phosphate receptor 1
6871139	1.23	0.0313	Mtvr2	mammary tumor virus receptor 2
6934130	1.23	0.0408	Arpc3	actin related protein 2/3 complex, subunit 3
6813172	1.24	0.0423	Shc3	src homology 2 domain-containing transforming protein C3
6759729	1.24	0.0183	Aamp	angio-associated migratory protein
6822852	1.25	0.0490	Lrrc3b	leucine rich repeat containing 3B
6848947	1.26	0.0454	Lix1	limb expression 1 homolog (chicken)
7011602	1.27	0.0453	Ldoc1	leucine zipper, down-regulated in cancer 1
6993131	1.27	0.0469	Sacm1l	SAC1 (suppressor of actin mutations 1, homolog)-like (S. cerevisiae)
7013294	1.34	0.0054	Pou3f4	POU domain, class 3, transcription factor 4
6868404	1.59	0.0135	Rfk	riboflavin kinase

Supplementary Table S2. Primers for genotyping and Quantitative RT-PCR

Rfk (NM_019437) Forward	CCCCACAGCCAATTTTCCTGA
Rfk (NM_019437) Reverse	TGCTCACCACCATTTTATGGAC
KCNIP2 (NM_145704) Forward	GGATGAGTTTGAACTATCCACGG
KCNIP2 (NM_145704) Reverse	GACAATTCCGCTGGGACATTC
hmTDP Forward	AAGGAATTCTGCATGCCCCAGATGCTGGCT
hTDP43 Reverse	CTGCTATTACCAAATCCACCCTGATTC
mTDP43 Reverse	GCCAGCTTCACATTTAAATTCATGCTCC
